# The effect of COVID-19 vaccine to the Omicron variant in children and adolescents: a systematic review and meta-analysis

**DOI:** 10.3389/fpubh.2024.1338208

**Published:** 2024-04-10

**Authors:** Wenting Lu, Shuai Zeng, Yuan Yao, Yiting Luo, Tiechao Ruan

**Affiliations:** ^1^Institute of Respiratory Health and Multimorbidity, West China Hospital, Sichuan University, Chengdu, Sichuan, China; ^2^Integrated Care Management Center, West China Hospital, Sichuan University, Chengdu, Sichuan, China; ^3^Department of Obstetrics and Gynecology, National Clinical Research Center for Obstetrics and Gynecology (Peking University Third Hospital), National Center for Healthcare Quality Management in Obstetrics, Peking University Third Hospital, Peking University, Beijing, China; ^4^General Practice Ward/International Medical Center Ward, General Practice Medical Center, West China Hospital, Sichuan University, Chengdu, China; ^5^Department of Pediatrics, West China Second University Hospital, Sichuan University, Chengdu, China; ^6^Key Laboratory of Obstetrics & Gynecologic and Pediatric Diseases and Birth Defects of the Ministry of Education, Sichuan University, Chengdu, China

**Keywords:** SARS-CoV-2 variants, Omicron, COVID-19 vaccines, child, adolescent

## Abstract

**Background:**

Omicron (B.1.1.529), a variant of SARS-CoV-2, has emerged as a dominant strain in COVID-19 pandemic. This development has raised concerns about the effectiveness of vaccination to Omicron, particularly in the context of children and adolescents. Our study evaluated the efficacy of different COVID-19 vaccination regimens in children and adolescents during the Omicron epidemic phase.

**Methods:**

We searched PubMed, Cochrane, Web of Science, and Embase electronic databases for studies published through March 2023 on the association between COVID-19 vaccination and vaccine effectiveness (VE) against SARS-CoV-2 infection in children and adolescents at the Omicron variant period. The effectiveness outcomes included mild COVID-19 and severe COVID-19. This study followed the Preferred Reporting Items for Systematic Reviews and Meta-Analyses guidelines and was prospectively registered in PROSPERO (CRD42023390481).

**Results:**

A total of 33 studies involving 16,532,536 children were included in the analysis. First, in children and adolescents aged 0–19 years, the overall VE of the COVID-19 vaccine is 45% (95% confidence interval [CI]: 40 to 50%). Subgroup analysis of VE during Omicron epidemic phase for different dosage regimens demonstrated that the VE was 50% (95% CI: 44 to 55%) for the 2-dose vaccination and 61% (95% CI: 45 to 73%) for the booster vaccination. Upon further analysis of different effectiveness outcomes during the 2-dose vaccination showed that the VE was 41% (95% CI: 35 to 47%) against mild COVID-19 and 71% (95% CI: 60 to 79%) against severe COVID-19. In addition, VE exhibited a gradual decrease over time, with the significant decline in the efficacy of Omicron for infection before and after 90 days following the 2-dose vaccination, registering 54% (95% CI: 48 to 59%) and 34% (95% CI: 21 to 56%), respectively.

**Conclusion:**

During the Omicron variant epidemic, the vaccine provided protection against SARS-CoV-2 infection in children and adolescents aged 0–19 years. Two doses of vaccination can provide effective protection severe COVID-19, with booster vaccination additionally enhancing VE.

## 1 Introduction

Since the emergence of severe acute respiratory syndrome coronavirus 2 (SARS-CoV-2) in 2019, its global impact has been profound (1), causing millions of infections and significantly affecting both human lives and socio-economic stability (2). As the epidemic evolves, the Omicron variant became the predominant strain of novel coronavirus pneumonia worldwide since November 2021 (3, 4). With the emergence of Omicron variant, the incidence of SARS-CoV-2 infections is growing among children (5), including mild COVID-19 (fever, fatigue, persistent dry cough, decreased or loss the sense of taste or smell and other symptoms) and severe COVID-19 (pneumonia, or life-threatening complications affecting the gastrointestinal, neurological, cardiovascular systems, or hospitalizations) (6).

Vaccination is the most economically efficient means to guard against COVID-19 (7, 8). And the efficacy of vaccination is linked to the vaccination dosages and the vaccination interval (9–13). The vaccination regimen currently comprises complete vaccination (two doses), and the booster vaccination (three doses) in children (14, 15). Comprehending the efficacy of vaccines in children is crucial for informed decision-making regarding vaccine policies, including the necessity, timing, and dosages of vaccination for children. Piechottal et al. found that in children aged 5–11 years, mRNA vaccines are moderately effective against infections with the omicron variant and protect well against COVID-19 hospitalizations (16). However, there remains limited understanding regarding the protective efficacy of vaccines against the omicron variant infection in children of a wider age range. In addition, it is unclear that the reasonable time interval after the administration of two doses vaccination and the efficacy of vaccination in preventing both mild and severe infections among individuals aged 0–19 years. Therefore, we conducted the meta-analysis to explore the efficacy of COVID-19 vaccine in children and adolescents aged 0–19 years during Omicron epidemic phase.

The study explored the association between vaccine effectiveness (VE) of COVID-19 vaccine and children SARS-CoV-2 infections during the Omicron variant outbreak. Additional, subgroup analyses were conducted to identify potential factors including various vaccination dosages, diverse SARS-CoV-2 outcomes, and different time intervals after the two doses vaccination. The findings provided a reference for the vaccination strategy of children against COVID-19 during the Omicron variant period and offered robust support for safeguarding the health and safety of the pediatric population.

## 2 Methods

### 2.1 Registration

The present investigation adhered to the guidelines outlined in the Preferred Reporting Items for Systematic Reviews and Meta-Analyses (PRISMA) and was prospectively registered in PROSPERO under the registration number CRD42023390481. Initially, the PROSPERO protocol was designed to evaluate the effectiveness of various vaccine types. However, the most articles meeting our inclusion criteria focused on the BNT162b2 vaccine, with limited data available for other vaccine types. Consequently, we modified the protocol to explore the vaccine efficacy concerning dosage, infection severity, and vaccination intervals.

### 2.2 Information sources and search strategies

We conducted comprehensive systematic literature searches utilizing the PubMed, Cochrane, Web of Science, and Embase electronic databases/platforms, spanning until Feb. 2023. A structured search strategy was meticulously devised, encompassing pertinent Medical Subject Headings (MeSH) search terms such as “COVID-19 Vaccines,” and text words such as “COVID19 Virus Vaccines,” “Coronavirus Disease 2019 Vaccine.” As well as Supplementary Concept “SARS-CoV-2 variants,” or text words like “Omicron,” “SARS-CoV-2 BA.5 variant,” “COVID-19 Virus variant B.1.1.529,” and “SARS-CoV-2 omicron variant.” Moreover, we included MeSH term “Child,” or text words like “Child,” “Children,” and MeSH term “Pediatrics,” with corresponding text words “pediatric.” To ensure comprehensive coverage, we adapted the search strategy accordingly for the other electronic databases employed. The specific search strategy for each database/platform is shown in the Supplementary materials. Additionally, we meticulously examined the reference lists of the included studies to identify further relevant literature for inclusion.

### 2.3 Eligibility criteria

We conducted a systematic review of studies that investigated the effectiveness of COVID-19 vaccines in preventing Omicron variant infections among children and adolescents. Our study population comprised individuals aged 0–19 years, with no restrictions on vaccine types or dosages administered. For precise analysis, included studies must explicitly specify COVID-19 infection attributed to the Omicron variant (PCR-confirmed or antigen-test confirmed) as the outcome measure and provide accessible data on VE. Our study excluded reviews, case series, case reports, and studies involving non-human subjects.

### 2.4 Study selection process

A single investigator conducted the initial database search and diligently screened for any duplicate entries. Following the elimination of duplicates, two reviewers (TR and WL) meticulously evaluated the titles and abstracts of all records, subsequently scrutinizing the full texts of the eligible articles.

### 2.5 Data collection

Data pertaining to study design and methodology, author names, publication year, study location, sample size, age range, dosages of vaccination, different outcomes of SARS-CoV-2 infection during Omicron-dominant period, and potential confounding variables were meticulously extracted from the incorporated studies. The extraction process was carried out by two independent reviewers (TR and WL).

### 2.6 Study risk of bias assessment

The risk of bias for all chosen studies was independently evaluated by two reviewers (TR and WL) utilizing the Newcastle-Ottawa Scale (NOS) score. Subsequently, the quality of each study was categorized into three grades: low (0–3), moderate (4–6), and high (7–9).

### 2.7 Statistical analysis

Data from the including studies were meticulously extracted into Microsoft Excel and then imported into Stata 12 software (Stata Corp) and Review Manager 5.3 for conducting the meta-analyses. VE is defined as the reduction in disease incidence among vaccinated individuals compared to unvaccinated individuals. The VE and its accompanying 95% confidence intervals (CIs) were computed utilizing either adjusted or unadjusted risk ratios (RR): VE = (1 - RR) × 100%. The VE expressed in percentage values exceeding 0% indicate a potential protective impact of the vaccine. We employed pooled RR and VE to evaluate the correlation between COVID-19 vaccination in children and adolescents and SARS-CoV-2 infections during the Omicron-dominant period. To quantify inconsistency across studies and ascertain the percentage of variability in effect estimates potentially arising from heterogeneity rather than sampling error, the I^2^ statistic and Q test were used to evaluate each study heterogeneity. If the heterogeneity was significant and I^2^ > 50%, a random effects model was used; otherwise, a fixed effects model was used. *p* < 0.05 was considered statistically significant. Additionally, sensitivity analysis was performed to assess the robustness of associations by excluding one study at a time. To gauge publication bias, a funnel plot was constructed, and Egger’s and Begg’s tests were conducted.

Furthermore, we performed subgroup analyses by stratifying the different vaccination dosages, varying time intervals after the 2-dose vaccination, and distinct outcome of SARS-CoV-2 infections. Based on the information provided in the original studies, the dosages of vaccination were categorized into three subgroups: one dose indicating incomplete vaccination, two doses representing complete vaccination, and three doses administered as booster vaccination. The classification of outcomes was divided into two subgroups: mild COVID-19 (fever, fatigue, persistent dry cough, decreased or loss the sense of taste or smell and other symptoms) and severe COVID-19 (pneumonia, or life-threatening complications affecting the gastrointestinal, neurological, cardiovascular systems, or hospitalization) based on the outcome indicators reported in the original study data. The time intervals were divided into two subgroups, as reported in the original studies: ≤90 days and > 90 days following the two doses vaccination.

## 3 Results

**Figure 1:**
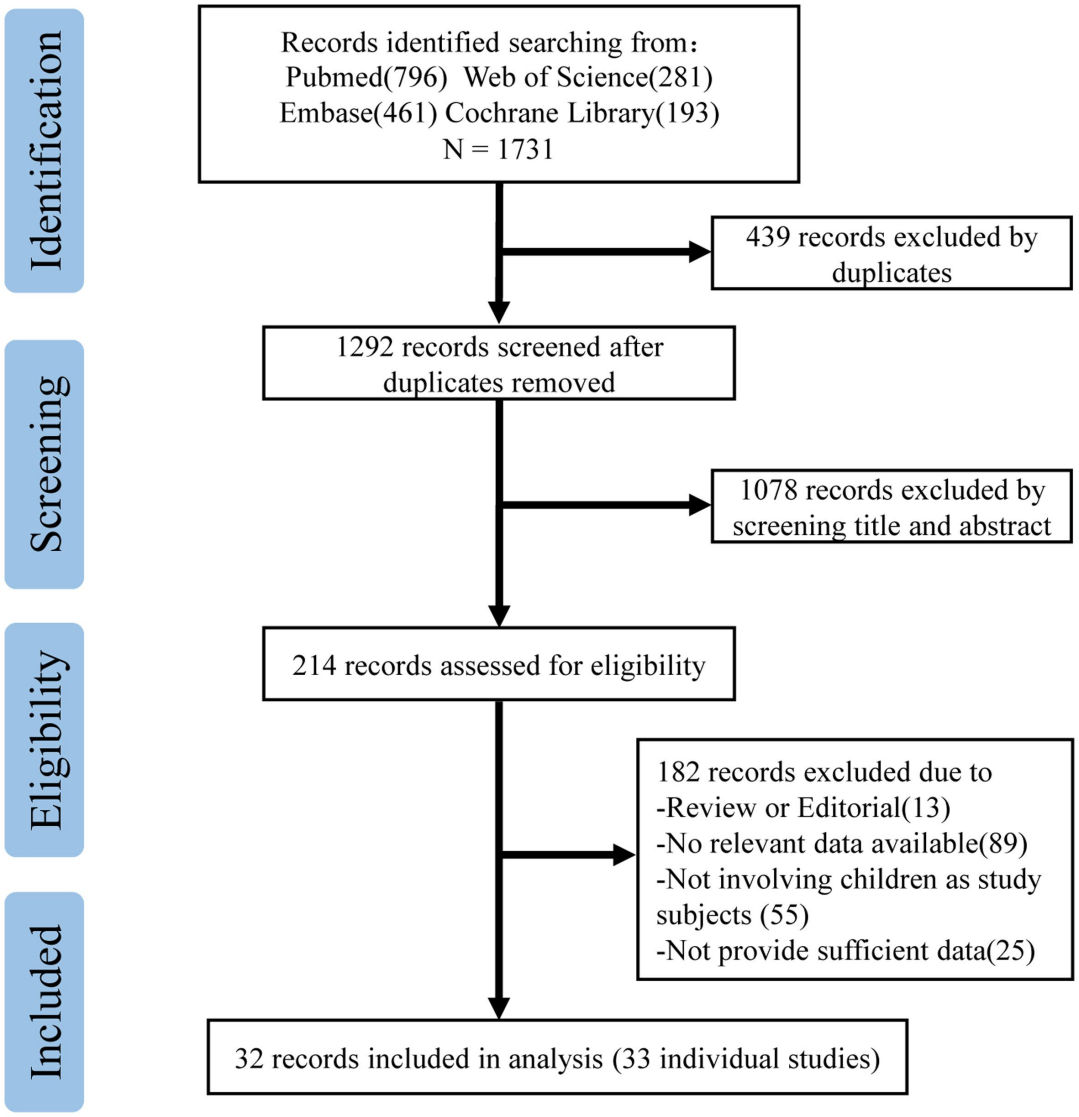
PRISMA flowchart.

The study selection process is shown in Figure 1. In our study, a total of 1731 records were searched in the databases to explore the efficacy of the COVID-19 vaccine in children aged 0–19 years during the Omicron-dominant period. In the course of our initial literature search and screening process, a total of 214 records underwent full-text evaluation. Among these, 13 records were recognized as reviews or editorials, 89 records lacked pertinent or valuable data, 55 records did not involve children or adolescents, and 25 records were unavailable in full text. Consequently, 32 records were eligible for inclusion in our study (Supplementary Table 1). Among these, we identified 15 cohort studies (10, 17–30) and 18 case–control studies (11, 12, 31–45), all utilizing non-vaccination as a control group, comprising an expansive cohort of 17,177,822 individuals. Of the 33 studies (one record contains two studies) included, 29 (87.88%) evaluated the effectiveness of the BNT162b2 vaccine, six (18.18%) involved the efficacy of the CoronaVac vaccine, two each on the effectiveness of the mRNA-1273 and the BBIBP-CorV vaccine, and one on the ChAdOx1nCoV-19 vaccine.

The NOS scores indicated that all the studies included in the analysis demonstrated moderate to high methodological quality. Among them, 17 studies (10, 17–19, 23–26, 28, 29, 32, 34, 35, 38, 43, 45) were rated as high quality, while 16 studies (11, 12, 20–22, 27, 30, 31, 33, 36, 37, 39–42, 44) were considered to be of medium quality (Supplementary Table 2).

**Figure 2:**
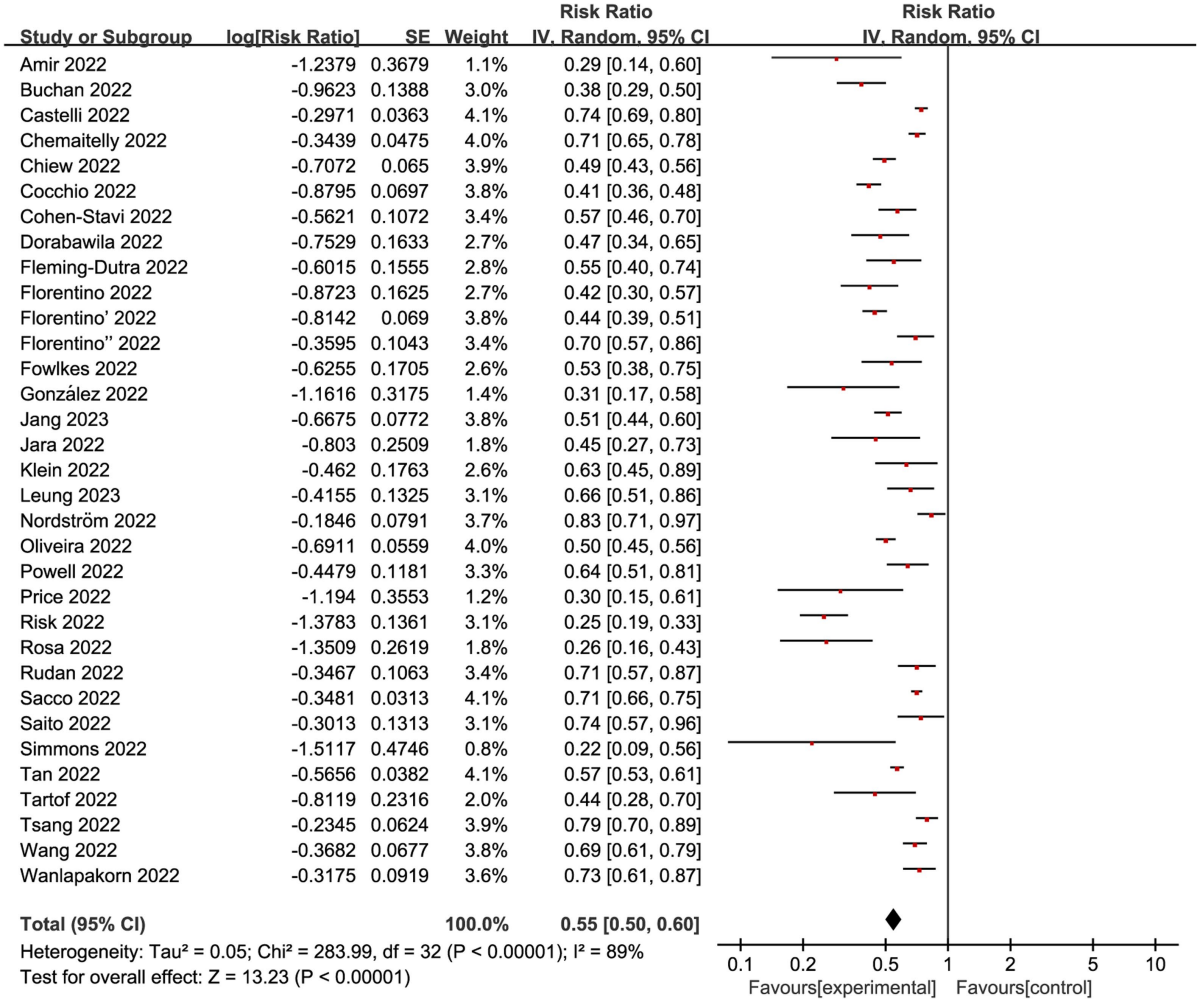
Forest plot for risk ratios on preventing Omicron infections. The red square symbolizes the point estimate for each study, with its size proportional to the study’s weight relative to the summary estimate. The black diamond symbol represents the overall effect estimate derived from the meta-analysis. Meta-analysis based on Random Effects model, inverse variance method (IV). Effect size estimates expressed in Log Risk Ratio [95%CI].

We next conducted a meta-analysis on 33 studies with eligible data to explore the VE for COVID-19 vaccine among children in Omicron-dominant period. The overall RR was 0.55 (95% CI: 0.50 to 0.60, I^2^ = 89%, *p* < 0.01; Figure 2; VE: 45, 95% CI: 40 to 50%; Table 1). Moreover, we evaluated the possibility of publication bias. The funnel plot resembles an asymmetrical distribution (Supplementary Figure 1). Egger’s test (*p* = 0.01; Supplementary Figure 2) and Begg’s test (*p* = 0.04; Supplementary Figure 3) showed publication bias. Therefore, we employed the trim-and-fill method to address publication bias, and we found that the results remained statistically significant after applying the trim-and-fill method (Supplementary Figure 4). This indicates the stability and reliability of our results, further supporting the validity of our conclusions. Then we performed sensitivity analysis indicated that the results were robust through removing a single study each time (Supplementary Figure 5).

**Table 1:** Overall effectiveness and vaccine effectiveness results of different vaccination regimens.

Outcome	No. Study	VE (95%CI) percent	Pooled RR (95%CI)	I^2^
SARS-CoV-2 Infection	33	45 (40, 50)	0.55 (0.50, 0.60)	89%
Different dosages				
1-dose	14	23 (18, 27)	0.77 (0.73, 0.82)	75%
2-dose	32	50 (44, 55)	0.50 (0.45, 0.56)	93%
3-dose	10	61 (45, 73)	0.39 (0.27, 0.55)	97%
Different dosages for BNT162b2 Vaccine				
1-dose	11	22 (17, 27)	0.78 (0.73, 0.83)	72%
2-dose	28	50 (44, 55)	0.50 (0.45, 0.56)	94%
3-dose	6	62 (45, 73)	0.38 (0.27, 0.55)	95%
Different dosages for CoronaVac Vaccine				
1-dose	5	14 (1, 25)	0.86 (0.75, 0.99)	14%
2-dose	6	52 (30, 66)	0.48 (0.34, 0.70)	90%
3-dose	1	38 (24, 49)	0.62 (0.51, 0.76)	-
2-dose Vaccination				
Different outcomes				
mild COVID-19	23	41 (35, 47)	0.59 (0,53, 0,65)	90%
severe COVID-19	12	71 (60, 79)	0.29 (0.21, 0.40)	92%
Time Intervals				
< 90d	21	54 (48, 59)	0.46 (0.41, 0.52)	85%
> 90d	15	34 (21, 45)	0.66 (0.55, 0.79)	91%
Mild COVID-19				
< 90d	20	51 (45, 56)	0.49 (0.44, 0.55)	85%
> 90d	13	25 (15, 35)	0.75 (0.65, 0.85)	93%
Severe COVID-19				
< 90d	5	76 (65, 84)	0.24 (0.16, 0.35)	79%
> 90d	5	56 (28, 73)	0.44 (0.27, 0.72)	95%

VE, Vaccine effectiveness; RR, Risk ratio; SARS-CoV-2, severe acute respiratory syndrome coronavirus 2; COVID-19, coronavirus disease 2019.

Additionally, subgroup analysis was conducted to identify potential factors that may influence the relationship between children’s vaccination and vaccine efficacy in preventing infections during the Omicron-dominant period. These factors included the dosages of vaccination, the classification of outcomes, and the interval between vaccine dosages.

**Figure 3:**
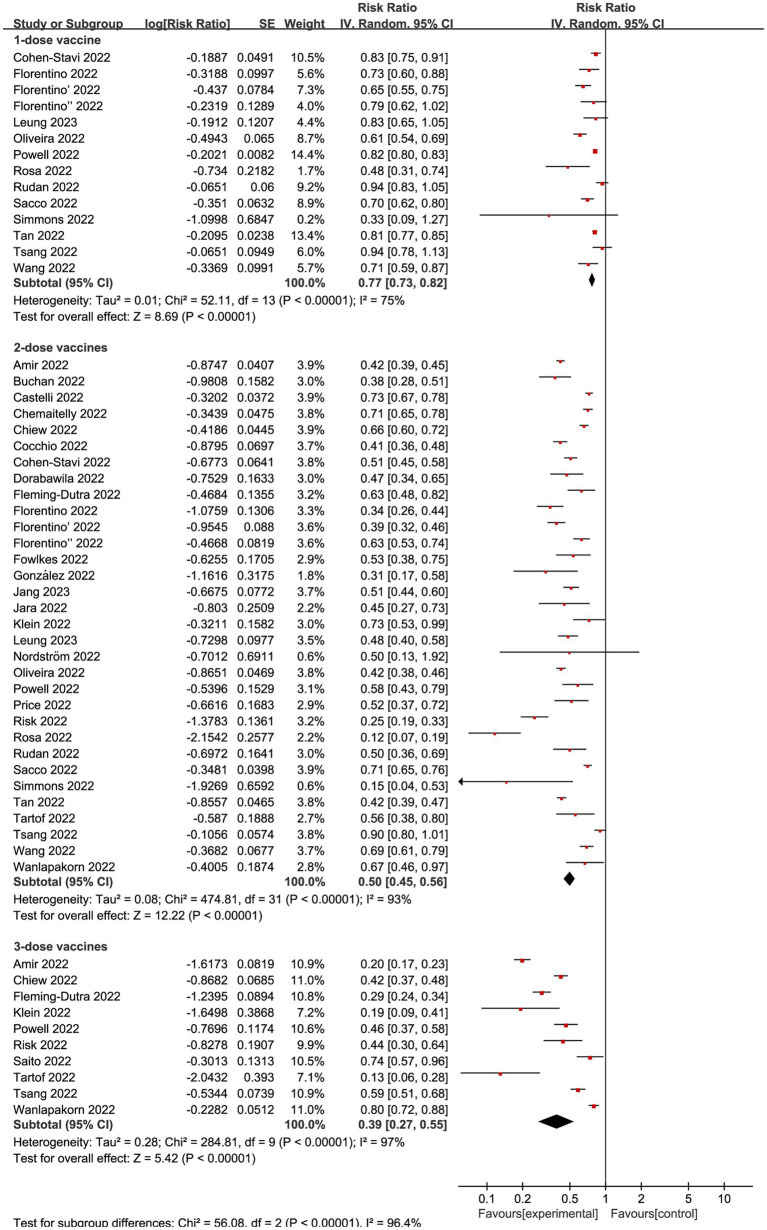
Forest plot for risk ratios of different vaccines dosages on preventing Omicron infections. The red square symbolizes the point estimate for each study, with its size proportional to the study’s weight relative to the summary estimate. The black diamond symbol represents the overall effect estimate derived from the meta-analysis. Meta-analysis based on Random Effects model, inverse variance method (IV). Effect size estimates expressed in Log Risk Ratio [95%CI].

Regardless of vaccination type, 14 studies (20, 26, 27, 29, 34, 37–41, 43–45) investigated VE of incomplete vaccination (1-dose) compared to non-vaccination individuals, revealing an overall RR of 0.77 (95% CI: 0.73 to 0.82, I^2^ = 75%, *p* < 0.01; Figure 3; VE: 23, 95% CI: 18 to 27%; Table 1). And a total of 32 studies (10–12, 17–41, 43–45) explored complete vaccination (2-dose), yielding an overall RR of 0.50 (95% CI: 0.45 to 0.56, I^2^ = 93%, *p* < 0.01; Figure 3; VE: 50, 95% CI: 44 to 55%; Table 1). Additionally, 10 studies (12, 17, 19, 25, 28–30, 36, 39, 42) focused on booster vaccination (3-dose), presenting an overall RR of 0.39 (95% CI: 0.27 to 0.55, I^2^ = 97%, *p* < 0.01; Figure 3; VE: 61, 95% CI: 45 to 73%; Table 1).

For the VE of vaccine dosages among different vaccine types (BNT162b2 and CoronaVac), we performed a subgroup analysis. 11 studies analyzed VE of 1-dose BNT162b2 vaccine, revealing an overall RR of 0.78 (95% CI: 0.73 to 0.83, I^2^ = 72%, *p* < 0.01; Supplementary Figure 6; VE: 22, 95% CI: 17 to 27%; Table 1) compared to non-vaccination individuals. And a total of 28 studies explored 2-dose BNT162b2 vaccine compared to non-vaccination individuals, yielding an overall RR of 0.50 (95% CI: 0.45 to 0.56, I^2^ = 94%, *p* < 0.01; Supplementary Figure 6; VE: 50, 95% CI: 44 to 55%; Table 1). Additionally, seven studies focused on 3-dose BNT162b2 vaccine, presenting an overall RR of 0.38 (95% CI: 0.27 to 0.55, I^2^ = 95%, *p* < 0.01; Supplementary Figure 6; VE: 62, 95% CI: 45 to 73%; Table 1).

The effectiveness of the CoronaVac vaccine with 1-dose was investigated in five studies, demonstrating an overall RR of 0.86 (95% CI: 0.75 to 0.99, I^2^ = 14%, *p* = 0.32; Supplementary Figure 7; VE: 14, 95% CI: 1 to 25%; Table 1). For the 2-dose regimen, a total of six studies were evaluated, revealing an overall RR of 0.48 (95% CI: 0.34 to 0.70, I^2^ = 90%, *p* < 0.01; Supplementary Figure 7; VE: 52, 95% CI: 30 to 66%; Table 1). In addition, only one study looked at the efficacy of 3-dose CoronaVac vaccine, yielding a RR of 0.62 (95% CI: 0.51 to 0.76; Supplementary Figure 7; VE: 38, 95% CI: 24 to 49%; Table 1).

**Figure 4:**
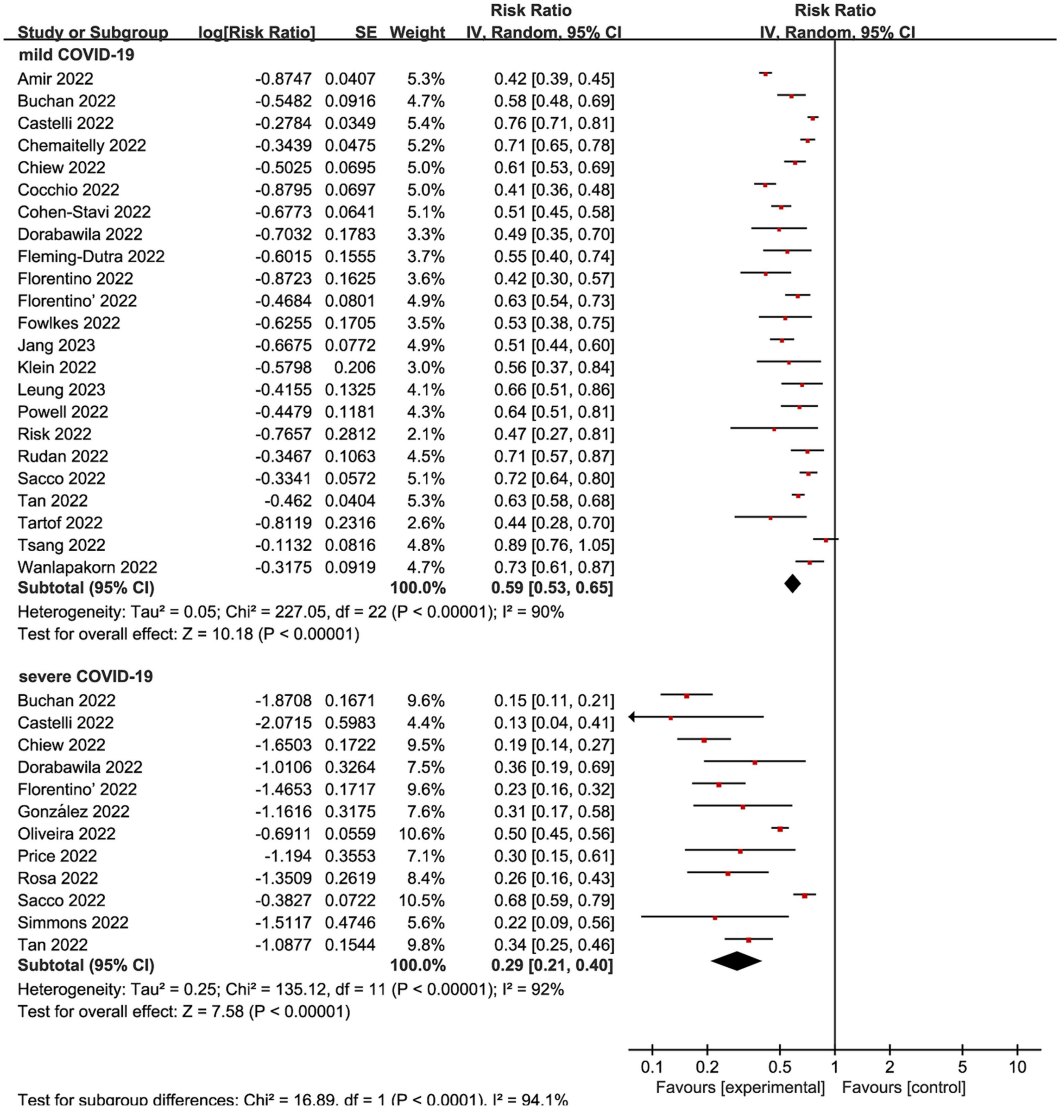
Forest plot for risk ratios on various outcomes following Omicron infections. The red square symbolizes the point estimate for each study, with its size proportional to the study’s weight relative to the summary estimate. The black diamond symbol represents the overall effect estimate derived from the meta-analysis. Meta-analysis based on Random Effects model, inverse variance method (IV). Effect size estimates expressed in Log Risk Ratio [95%CI].

We subsequently conducted subgroup analyses within the complete vaccination group, focusing on distinct outcome measures. Nine studies (12, 20, 21, 26, 29, 31, 34, 39, 45) made mild COVID-19 as the outcome, presenting an overall RR of 0.59 (95% CI: 0.53 to 0.65, I^2^ = 90%, *p* < 0.01; Figure 4; VE: 41, 95% CI:35 to 47%; Table 1). Meanwhile, complete vaccination could decrease the risks of Omicron associated severe COVID-19 with RR of 0.29 (95% CI: 0.21 to 0.40, I^2^ = 92%, *p* < 0.01; Figure 4; VE: 71, 95% CI: 60 to 79%; Table 1).

**Figure 5:**
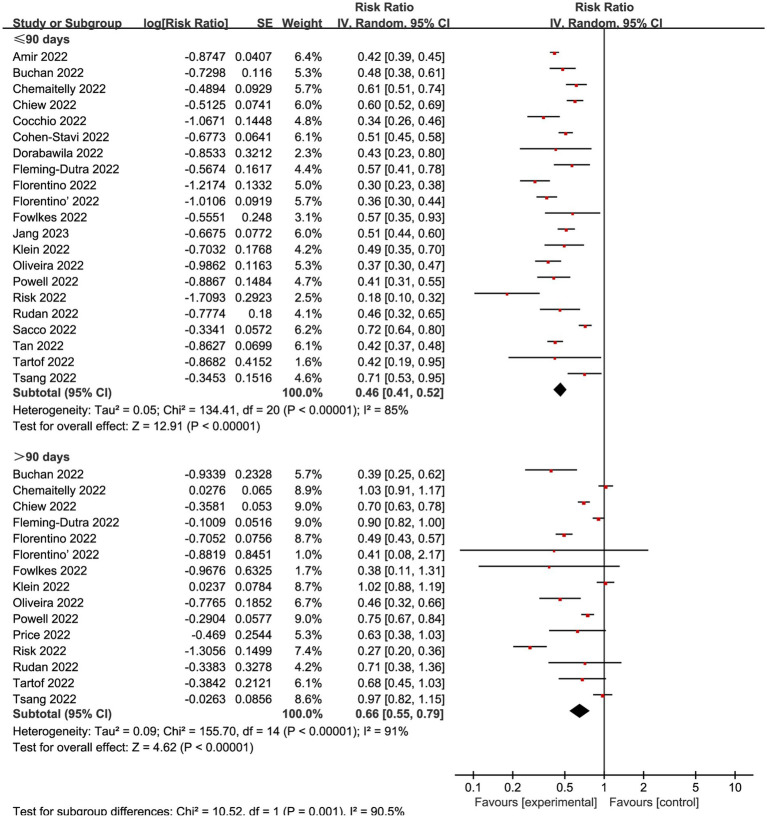
Forest plot for risk ratios of different time intervals after 2-dose vaccination on preventing Omicron infections. The red square symbolizes the point estimate for each study, with its size proportional to the study’s weight relative to the summary estimate. The black diamond symbol represents the overall effect estimate derived from the meta-analysis. Meta-analysis based on Random Effects model, inverse variance method (IV). Effect size estimates expressed in Log Risk Ratio [95%CI].

Many studies showed that the vaccine demonstrates its optimal protective effect within about 90 days following the second dose (13, 18, 46, 47). Therefore, we conducted subgroup analyses in the complete vaccinated group using 90 days as a reference time point. Among the 33 studies included, 23 studies incorporated time-based monitoring of outcome indicators, while the remaining 10 studies did not specify temporal conditions. When combining all VE evaluations of complete vaccination within 90 days, the vaccination decreased infection by an overall RR of 0.46 (95% CI: 0.41 to 0.52, I^2^ = 85%, *p* < 0.01; Figure 5; VE: 54, 95% CI: 48 to 59%; Table 1). The cumulative effectiveness of vaccination over 90 days after the complete vaccination was 0.66 (95% CI 0.55 to 0.79, I^2^ = 91%, *p* < 0.01; Figure 5; VE: 34, 95% CI: 21 to 45%; Table 1) in the vaccinated cohort.

**Figure 6:**
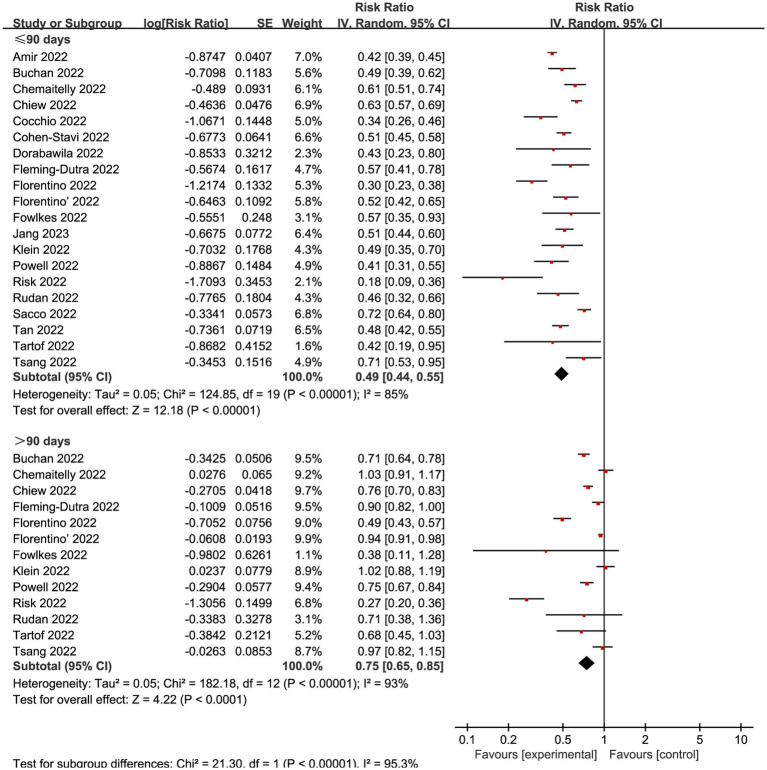
Forest plot for risk ratios of different time intervals after 2-dose vaccination on preventing mild COVID-19. The red square symbolizes the point estimate for each study, with its size proportional to the study’s weight relative to the summary estimate. The black diamond symbol represents the overall effect estimate derived from the meta-analysis. Meta-analysis based on Random Effects model, inverse variance method (IV). Effect size estimates expressed in Log Risk Ratio [95%CI].

**Figure 7:**
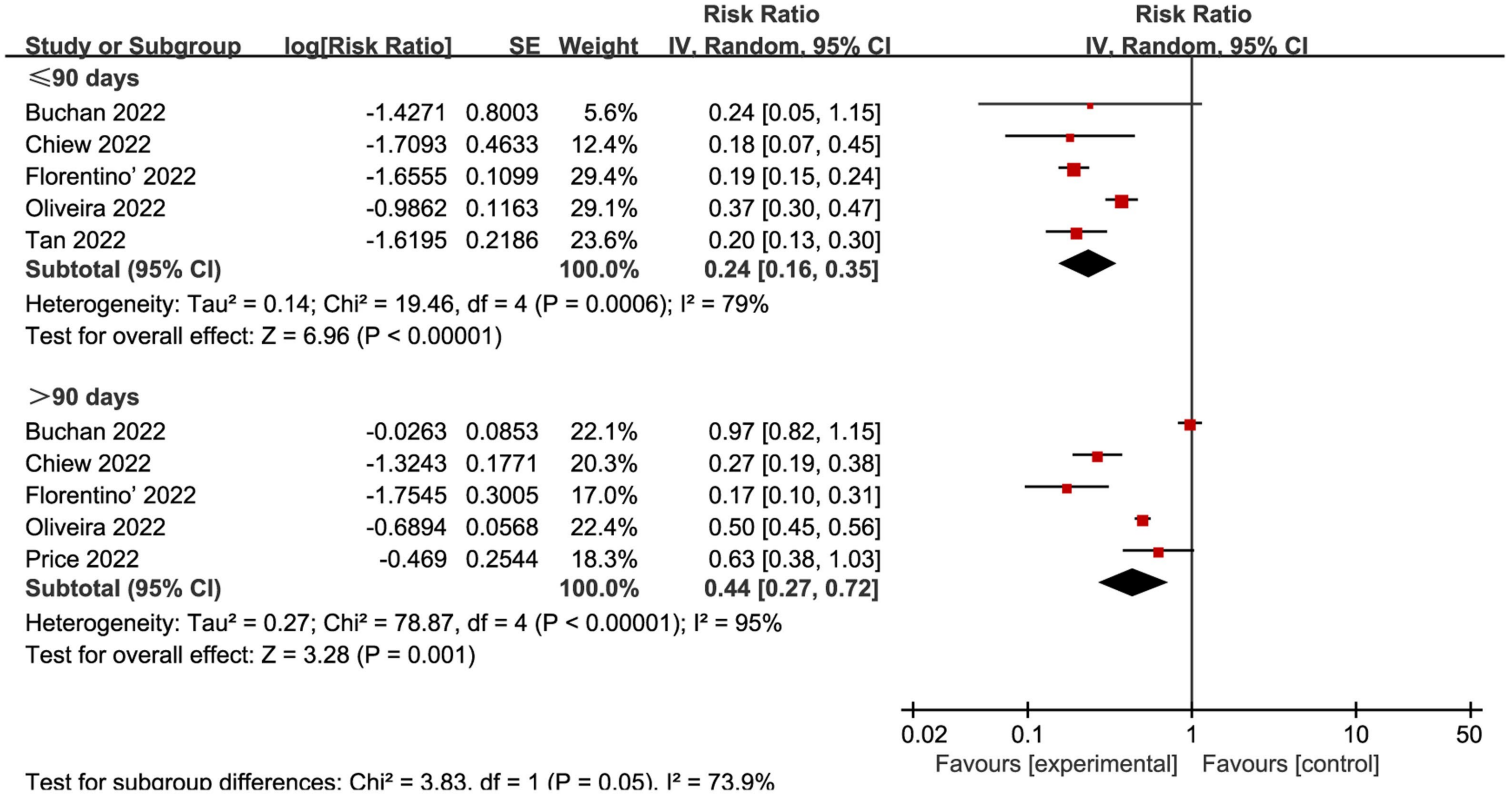
Forest plot for risk ratios of different time intervals after 2-dose vaccination on preventing severe COVID-19. The red square symbolizes the point estimate for each study, with its size proportional to the study’s weight relative to the summary estimate. The black diamond symbol represents the overall effect estimate derived from the meta-analysis. Meta-analysis based on Random Effects model, inverse variance method (IV). Effect size estimates expressed in Log Risk Ratio [95%CI].

We also explored the effects of vaccination at different time intervals across two outcomes. The VE against omicron mild COVID-19 in 90 days before and after were 0.49 (95% CI: 0.44 to 0.55, I^2^ = 85%, *p* < 0.01; Figure 6; VE: 51, 95% CI: 45 to 56%; Table 1) and 0.75 (95% CI: 0.65 to 0.85, I^2^ = 93%, *p* < 0.01; Figure 6; VE: 25, 95% CI: 15 to 35%; Table 1), respectively. Studies evaluated the VE, which decreased with time after receipt of the second dose, over time for the recent vaccination. As for the severe COVID-19, intervals less than 90 days or more than 90 days was associated with a decreased risk for Omicron with RR 0.24 (95% CI 0.16 to 0.35, I^2^ = 79%, *p* < 0.01; Figure 7; VE: 76, 95% CI: 65 to 84%; Table 1) and RR 0.44 (95% CI 0.27 to 0.72, I^2^ = 95%, *p* < 0.01; Figure 7; VE: 56, 95% CI: 28 to 73%; Table 1), respectively.

## 4 Discussion

The study focuses on the efficacy of COVID-19 vaccination among children and adolescents aged 0–19 during the Omicron-dominant period. The study shows that the vaccine provided protection against SARS-CoV-2 infections during the Omicron-dominant period. The VE trend is increasing with the additional booster vaccination regimen, and its efficacy varies for distinct SARS-CoV-2 infections within the 2-dose vaccination. The vaccine offered greater protection against severe COVID-19 during Omicron epidemic phase compared to mild COVID-19. A gradual decline in the efficacy of the COVID-19 vaccination against both mild and severe COVID-19 was observed over time, with a notable decline occurring after 90 days.

Our study estimates the VE against SARS-CoV-2 infections during Omicron-dominant phase was significantly lower than the VE during Delta-dominant phase (14, 48). The decline in VE during the Omicron-dominant period may be due to the increased incidence of breakthrough infections associated with the Omicron variant, along with the rapid and infectious transmission of this variant (49, 50). Furthermore, the enhanced potential of Omicron variant for immune evasion compared to the Delta variant may be involved in this phenomenon (51–53).

According to our study findings, there was a positive correlation between VE and the number of vaccinations administered. As the number of vaccinations increases, the VE gradually increases as well. It is well established that vaccine-induced immunity decreases over time (32). However, the increased dosages of the vaccine could maintain and generate favorable antibody, B-cell, and T-cell responses, thus providing robust protection to the body (54).

For vaccination intervals, some studies have suggested that a 3-month interval may be preferable to a vaccination program with the shorter intervals, which protects the largest number of individuals in the population as early as possible in case of supply shortages. The vaccination also improving protection after receiving the second dose (13). Furthermore, regulatory authorities in countries such as the United Kingdom have approved 2-dose intervals of up to 3 months for viral vector and mRNA vaccines (55, 56). The relative VE of the booster vaccination given more than 3 months after the second vaccination was 84.4% and the absolute VE for symptomatic SARS-CoV-2 infection was 94.0% in adults over 50 years of age compared with unvaccinated participants (57). However, some studies have found that the highest antibody response could occur in the first month after vaccination, but immunity declined rapidly in the next 3–4 months, with the peak antibody titers decreased by almost 4–5.5 times (58, 59). They supported that vaccine protection against Omicron variant infection waned within 3 months after the second dose, suggesting that a shorter interval between the second vaccination and booster may be beneficial (60). However, our study found no significant decline in VE for severe COVID-19 observed over the 3-month interval after the second vaccination. The most significant decline of VE was observed against mild COVID-19, where efficacy decreased by approximately 50% over the 3-month interval after second vaccination.

While COVID-19 vaccines provide steady protection against severe SARS-CoV-2 infections, their efficacy in preventing mild SARS-CoV-2 infections has been reduced, particularly during the Omicron-dominant period (61–63). It is well-established that antibodies or localized memory immune responses are the primary determinants of infection. COVID-19 vaccines primarily generate a systemic immune response, where immunoglobulin G (IgG) circulates in serum and body fluids as the primary functional component (64). With prophylactic vaccination, IgG antibodies remain in the serum for a certain duration. To prevent viral infection effectively, vaccine-induced serum IgG antibodies must enter the respiratory tract, coming into direct contact with lung endothelial cell surfaces to neutralize a viral infection (65, 66). However, due to a limited number of specific antibodies reaching the upper respiratory tract and gradual decreases in antibody concentrations over time (67), the COVID-19 vaccine’s immune response is ineffective to prevent virus replication in the upper respiratory tract. The reduction of local antibodies in the upper respiratory tract weakens the protective effect against antiviral infection, leading to decreased defense against mild COVID-19. However, the antibody library present in circulating blood in the lung efficiently blocks the virus from attacking the alveolar epithelium and capillary endothelium (68, 69), limiting severe pulmonary infections. In addition, Omicron cross-reactive T-cells and immune memory B-cells located throughout the body can be swiftly engaged upon encounter with a viral infection (70), producing an effective B and T cell-specific immune response (71). The immune memory B-cells produce large amounts of targeted antibodies to protect against the spread and replication of viruses, helping to prevent the onset of severe COVID-19 (72, 73). Accordingly, children who received complete vaccination during the Omicron-dominant period experienced a reduced risk of severe SARS-CoV-2 infection.

This meta-analysis has several merits. First, eligible studies were retrieved from current major literature databases to minimize the risk of omitting relevant studies. Second, all included studies were published after the emergence and spread of Omicron variants, and these data were representative of the Omicron epidemic period. Third, the research data of included studies was obtained from national electronic medical databases, providing a representative population sample and a large sample size. Finally, all included studies were of high or moderate methodological quality, providing high reliability for the meta-analysis.

Our study also had several limitations. Firstly, meta-analyses of VE show a high degree of heterogeneity. Although sensitivity and subgroup analyses were performed to identify possible sources of heterogeneity, it appears that heterogeneity was not because of the degrees of infection, dosages of vaccination, or vaccination intervals. The type of vaccination, the body’s immune response, and variations in population characteristics might be responsible for the heterogeneity. However, due to insufficient data available from the included studies to stratify these variations, identifying the source of heterogeneity was challenging. Secondly, the findings regarding VE against SARS-CoV-2 infections in children and adolescents during the Omicron-dominant period may exhibit minor bias. However, further studies with larger sample sizes are warranted. In addition, some of the included studies did not provide exact time after second vaccination to evaluation of VE. Therefore, the evaluation of efficacy at longer time nodes in this study is relatively limited. The VE at different time nodes is still uncertain. Although boosters may improve efficacy, the timing of boosters remain to be further investigated.

## 5 Conclusion

During the period dominated by the Omicron variant, vaccination has demonstrated its ability to reduce the risk of SARS-CoV-2 infection in children and adolescents aged 0 to 19 years. The effectiveness of the vaccine becomes more pronounced as the number of dosages increases. Two doses vaccination significantly reduces the risk of severe COVID-19. The protection was still present but decreased over 90 days after the second vaccine regimen.

## Author contributions

WL: Conceptualization, Formal analysis, Investigation, Methodology, Writing – original draft, Writing – review & editing. SZ: Data curation, Investigation, Writing – original draft. YY: Data curation, Investigation, Writing – original draft. YL: Data curation, Formal analysis, Writing – original draft. TR: Investigation, Methodology, Supervision, Writing – original draft, Writing – review & editing.
