# Supplementary material for: The effect of COVID-19 vaccine to the Omicron variant in children and adolescents: a systematic review and meta-analysis
Source: Front Public Health. 2024 Apr 10;12:1338208. doi: 10.3389/fpubh.2024.1338208 (PMC11041831; doi:10.3389/fpubh.2024.1338208)
Supplement: Supplementary file 1 [file Presentation_1.pdf]

Supplementary Material

Appendix 1: Search strategy

1.PubMed 02/06/2023 Results:796

((((("SARS-CoV-2 variants" [Supplementary Concept]) OR (Omicron)) OR (SARS-CoV-2 BA.5 variant)) OR (COVID-19 Virus variant B.1.1.529)) OR (SARS-CoV-2 omicron variant)) AND ((((((("COVID-19 Vaccines"[Mesh]) OR (Vaccine)) OR (COVID 19 Vaccines)) OR (COVID 19 Virus Vaccine)) OR (Coronavirus Disease 2019 Vaccine)) OR (SARS2 Vaccines)) OR (SARS CoV 2 Vaccine))) AND ((("Pediatrics"[Mesh]) OR (pediatric)) OR ((("Child"[Mesh]) OR (Children)) OR (Child)))

2.Web of Science 02/06/2023 Results:281

#1 (((TS=(Omicron)) OR TS=(SARS-CoV-2 BA.5 variant)) OR TS=(COVID-19 Virus variant B.1.1.529) OR TS=(SARS-CoV-2 omicron variant)

#2 ((((((TS=(COVID-19 Vaccines)) OR TS=(Vaccine)) OR TS=(COVID 19 Vaccines)) OR TS=(COVID 19 Virus Vaccine)) OR TS=(Coronavirus Disease 2019 Vaccine)) OR TS=(SARS2 Vaccines)) OR TS=(SARS COV 2 Vaccine)

#3 (((TS=(Child)) OR TS=(Children)) OR TS=(Pediatrics)) OR TS=(Pediatric)

#4 #1 AND #2 AND #3

3. Embase 02/06/2023 Results:461

#1 ‘child’/exp OR ‘child’ OR ‘children’

#2 ‘pediatric’/exp OR ‘pediatric’

#3 #1 OR #2

#4 ‘sars-cov-2 vaccine’/exp OR ‘covid-19 vaccines’/exp OR ‘vaccine’/exp OR ‘covid 19 vaccines’/exp OR ‘covid 19 virus vaccine’/exp OR ‘Coronavirus disease 2019 vaccine’/exp OR ‘sars2 vaccines’ OR ‘sars cov 2 vaccine’/exp

#5 ‘omicron’ OR ‘sars-cov-2 ba.5 variant’ OR ‘covid-19 virus variant b.1.1.529’ OR ‘sars-cov-2 omicron’/exp OR ‘sars-cov-2 omicrion’

#6 #3 AND #4 AND #5

4. Cochrane 02/06/2023 Results:193

#1 MeSH descriptor: [Child] explode all trees

#2 Children

#3 #1 OR #2

#4 MeSH descriptor:[Pediatrics] explode all trees

#5 Pediatric

#6 #4 OR #5

#7 #3 OR #6

#8 MeSH descriptor:[COVID-19 Vaccines] explode all trees

#9 Vaccine OR ‘COVID 19 Vaccines’ OR ‘COVID 10 Virus Vaccine’ OR ‘Coronavirus Disease 2019 Vaccine’ OR ‘SARS2 Vaccines’ OR ‘SARS CoV 2 Vaccine’

#10 #8 OR #9

#11 Omicron OR’SARS-CoV-2 BA.5 variant’ OR ‘COVID-19 Virus variant B.1.1.529’ OR ‘SARS-CoV-2 omicron variant’

#12 #7 AND #10 AND #11

frontiers

Appendix 2: Supplementary Figures

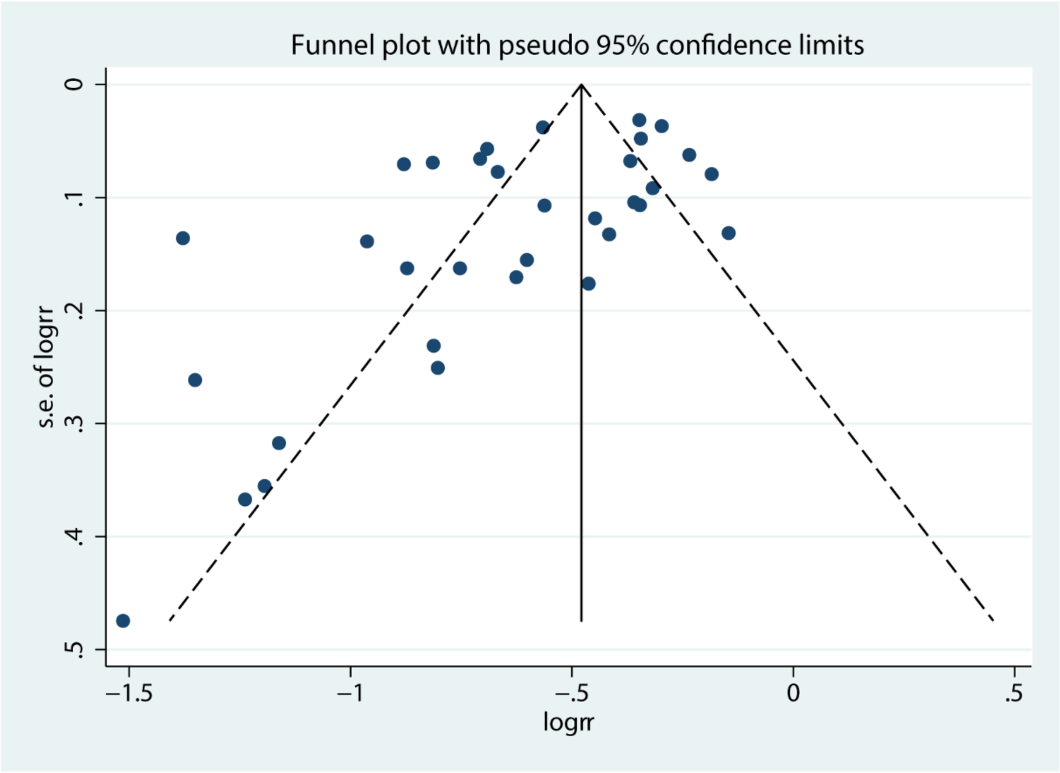

Supplemental Figure 1. Funnel plot of vaccine effectiveness studies. The funnel plots are centered at 0 (i.e., at the value under the null hypothesis of no effect) and display the studies' results (x-axis) and their precision (y-axis). In the meta-analyses, the result is expressed as log risk ratios (RRs) and the precision is represented by the standard error of the estimates. Each dot represents a single observation. The funnel plot shows an asymmetry indicating potential publication bias.

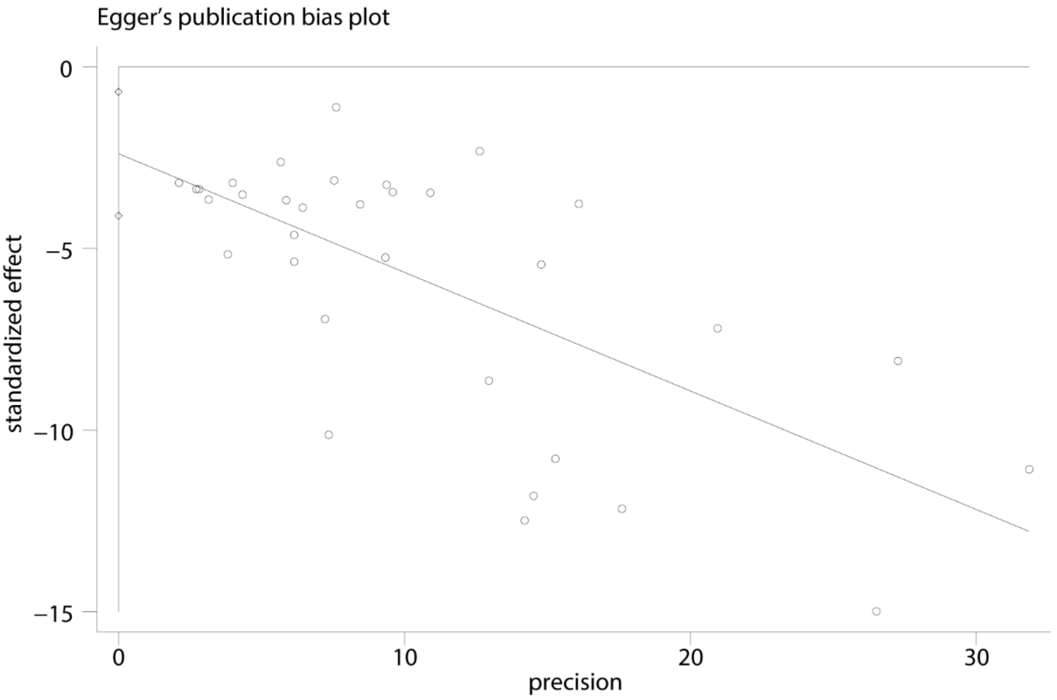

Supplemental Figure 2. Egger's publication bias plot of vaccine effectiveness studies. The X-axis represents the precision size, while the Y-axis depicts the standardized effect size. Each data point represents an individual study, with its position indicating the observed effect size and standard error. Studies deviating from the regression line suggesting potential publication bias (t value of Egger's test =-2.86, p=0.01).

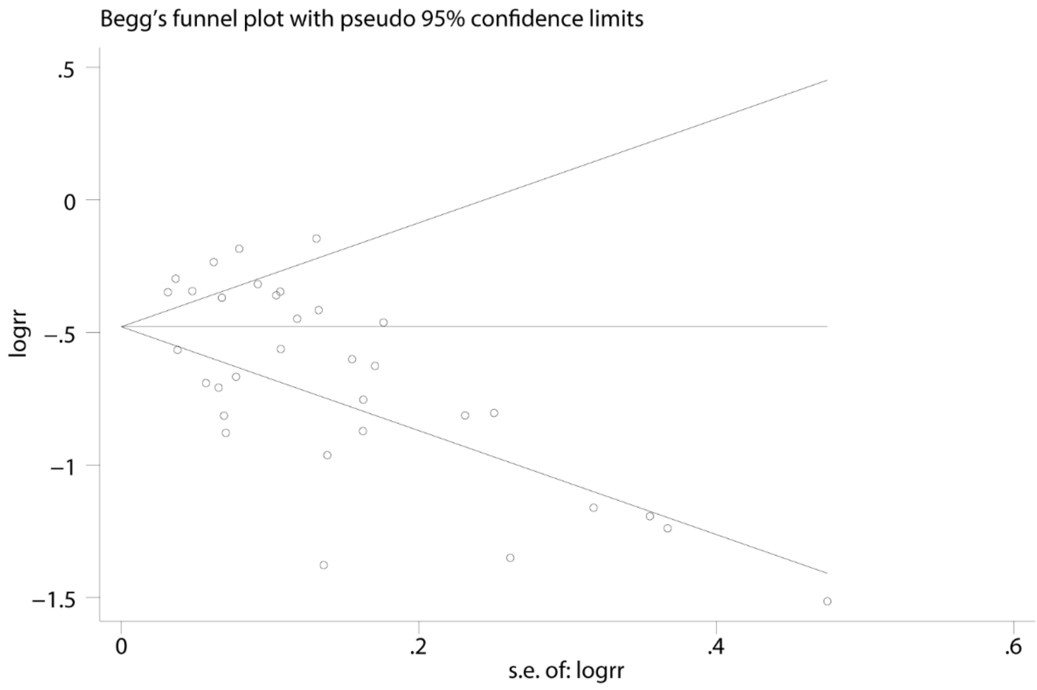

Supplemental Figure 3. Begg's funnel plot of vaccine effectiveness studies. The X-axis is expressed as log risk ratios (RRs) and the Y-axis depicts the standardized effect size. Each data point represents an individual study, with its position indicating the observed effect size and standard error. Smaller studies scattered widely at the bottom, and larger studies clustered closer to the top. Studies deviating from the regression line suggesting potential publication bias (z value of Begg's test =2.09, p=0.04).

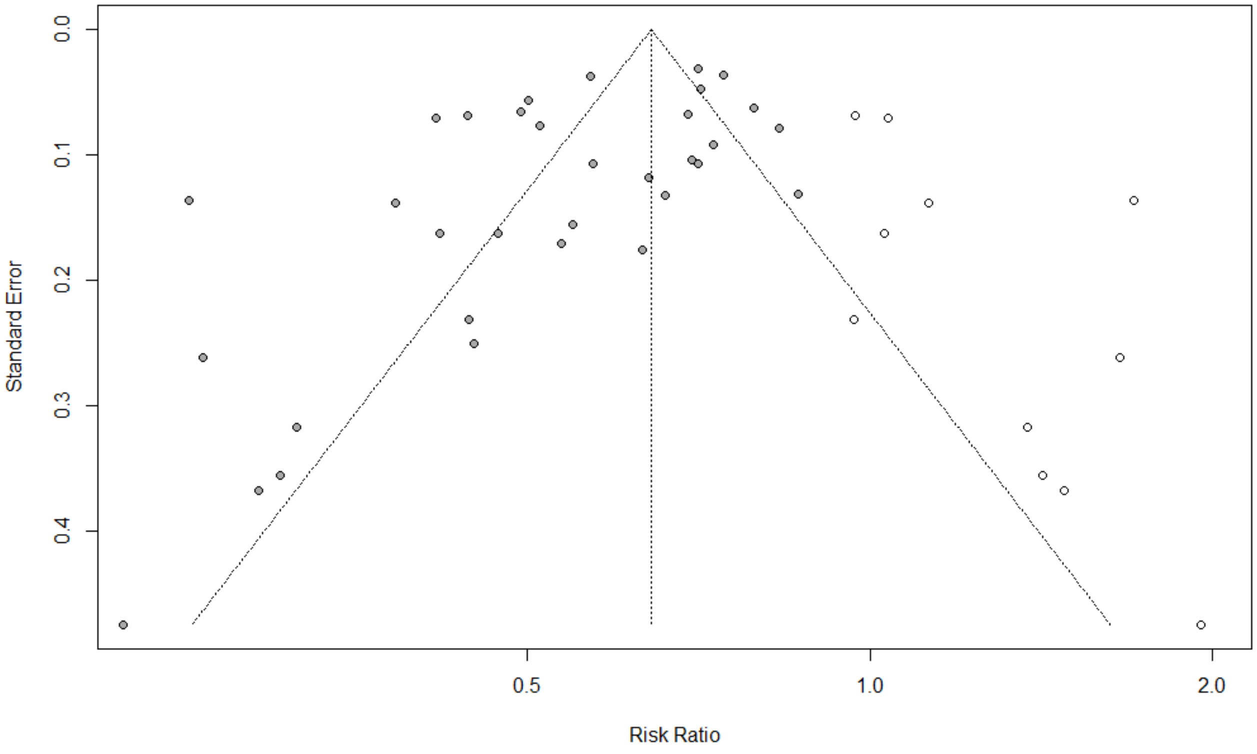

Supplemental Figure 4. Filled funnel plot of vaccine effectiveness studies. The filled funnel plot integrates black dots representing included studies and gray circles representing additional studies imputed through trim-and-fill analysis to address potential publication bias. The inclusion of 11 imputed studies in the trim-and-fill analysis suggests no significant publication bias after adjustment (t value of adjustment Egger's test =-0.38, p=0.71).

frontiers

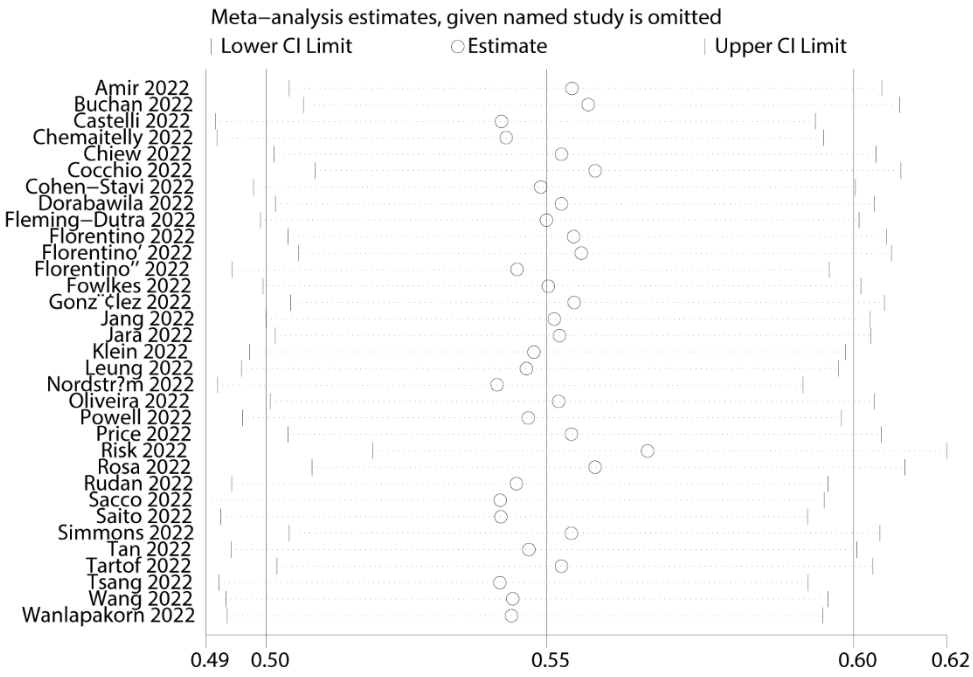

Supplemental Figure 5. The sensitive analysis of vaccine effectiveness studies. Each line or data point represents a different sensitivity analysis scenario, such as excluding certain studies or using different statistical models. When points cluster closer to the central reference line, it indicates greater robustness and consistency in the results across different scenarios, suggesting higher reliability. The results were robust through removing a single study each time.

Study or Subgroup	log[Risk Ratio]	SE	Weight	Risk Ratio IV, Random, 95% CI
**1-dose BNT162b2 vaccine**				
Cohen-Stavi 2022	-0.1886	0.0492	14.5%	0.83 [0.75, 0.91]
Florentino 2022	-0.3193	0.0994	7.3%	0.73 [0.60, 0.88]
Florentino' 2022	-0.4365	0.0786	9.7%	0.65 [0.55, 0.75]
Leung 2023	-0.3434	0.1197	5.6%	0.71 [0.56, 0.90]
Oliveira 2022	-0.4301	0.1466	4.1%	0.65 [0.49, 0.87]
Powell 2022	-0.2022	0.0081	21.2%	0.82 [0.80, 0.83]
Rosa 2022	-1.1992	0.2602	1.5%	0.30 [0.18, 0.50]
Rudan 2022	-0.0651	0.06	12.5%	0.94 [0.83, 1.05]
Simmons 2022	-1.0998	0.6847	0.2%	0.33 [0.09, 1.27]
Tan 2022	-0.2088	0.0242	19.2%	0.81 [0.77, 0.85]
Tsang 2022	-0.2111	0.1437	4.2%	0.81 [0.61, 1.07]
**Subtotal (95% CI)**			**100.0%**	**0.78 [0.73, 0.83]**
Heterogeneity: Tau² = 0.01; Chi² = 35.69, df = 10 (P < 0.0001); I² = 72%				
Test for overall effect: Z = 7.58 (P < 0.00001)				
**2-dose BNT162b2 vaccines**				
Amir 2022	-0.871	0.0426	4.6%	0.42 [0.39, 0.45]
Buchan 2022	-0.9831	0.1571	3.5%	0.37 [0.27, 0.51]
Castelli 2022	-0.3293	0.0199	4.7%	0.72 [0.69, 0.75]
Chemaitelly 2022	-0.3608	0.0256	4.7%	0.70 [0.66, 0.73]
Chiew 2022	-0.4186	0.0445	4.6%	0.66 [0.60, 0.72]
Cocchio 2022	-0.8313	0.0915	4.2%	0.44 [0.36, 0.52]
Cohen-Stavi 2022	-0.6773	0.0641	4.5%	0.51 [0.45, 0.58]
Dorabawila 2022	-0.6056	0.2194	2.8%	0.55 [0.36, 0.84]
Fleming-Dutra 2022	-0.4687	0.1354	3.7%	0.63 [0.48, 0.82]
Florentino 2022	-1.0775	0.1297	3.8%	0.34 [0.26, 0.44]
Florentino' 2022	-0.955	0.0877	4.2%	0.38 [0.32, 0.46]
Fowlkes 2022	-0.6257	0.1704	3.3%	0.53 [0.38, 0.75]
González 2022	-1.4389	0.4562	1.2%	0.24 [0.10, 0.58]
Jang 2023	-0.6673	0.0773	4.3%	0.51 [0.44, 0.60]
Klein 2022	-0.3211	0.1582	3.4%	0.73 [0.53, 0.99]
Leung 2023	-0.7976	0.1556	3.5%	0.45 [0.33, 0.61]
Oliveira 2022	-0.9012	0.1152	4.0%	0.41 [0.32, 0.51]
Powell 2022	-0.5394	0.153	3.5%	0.58 [0.43, 0.79]
Price 2022	-1.6693	0.3713	1.5%	0.19 [0.09, 0.39]
Risk 2022	-1.3784	0.1361	3.7%	0.25 [0.19, 0.33]
Rosa 2022	-2.1287	0.4632	1.1%	0.12 [0.05, 0.29]
Rudan 2022	-0.6971	0.1642	3.4%	0.50 [0.36, 0.69]
Sacco 2022	-0.3483	0.0397	4.6%	0.71 [0.65, 0.76]
Simmons 2022	-1.9269	0.6592	0.6%	0.15 [0.04, 0.53]
Tan 2022	-0.8562	0.0462	4.6%	0.42 [0.39, 0.47]
Tartof 2022	-0.5864	0.1891	3.1%	0.56 [0.38, 0.81]
Tsang 2022	-0.1126	0.0819	4.3%	0.89 [0.76, 1.05]
Wanlapakorn 2022	-0.227	0.0448	4.6%	0.80 [0.73, 0.87]
**Subtotal (95% CI)**			**100.0%**	**0.50 [0.45, 0.56]**
Heterogeneity: Tau² = 0.07; Chi² = 481.84, df = 27 (P < 0.00001); I² = 94%				
Test for overall effect: Z = 12.28 (P < 0.00001)				
**3-dose BNT162b2 vaccines**				
Amir 2022	-1.6173	0.0819	15.5%	0.20 [0.17, 0.23]
Buchan 2022	-0.9137	0.1216	14.9%	0.40 [0.32, 0.51]
Chiew 2022	-0.8682	0.0685	15.6%	0.42 [0.37, 0.48]
Powell 2022	-0.7696	0.1174	15.0%	0.46 [0.37, 0.58]
Saito 2022	-0.3013	0.1313	14.7%	0.74 [0.57, 0.96]
Tartof 2022	-2.0432	0.393	9.1%	0.13 [0.06, 0.28]
Tsang 2022	-0.6029	0.1078	15.1%	0.55 [0.44, 0.68]
**Subtotal (95% CI)**			**100.0%**	**0.38 [0.27, 0.55]**
Heterogeneity: Tau² = 0.21; Chi² = 112.72, df = 6 (P < 0.00001); I² = 95%				
Test for overall effect: Z = 5.29 (P < 0.00001)				
Test for subgroup differences: Chi² = 56.29, df = 2 (P < 0.00001), I² = 96.4%				

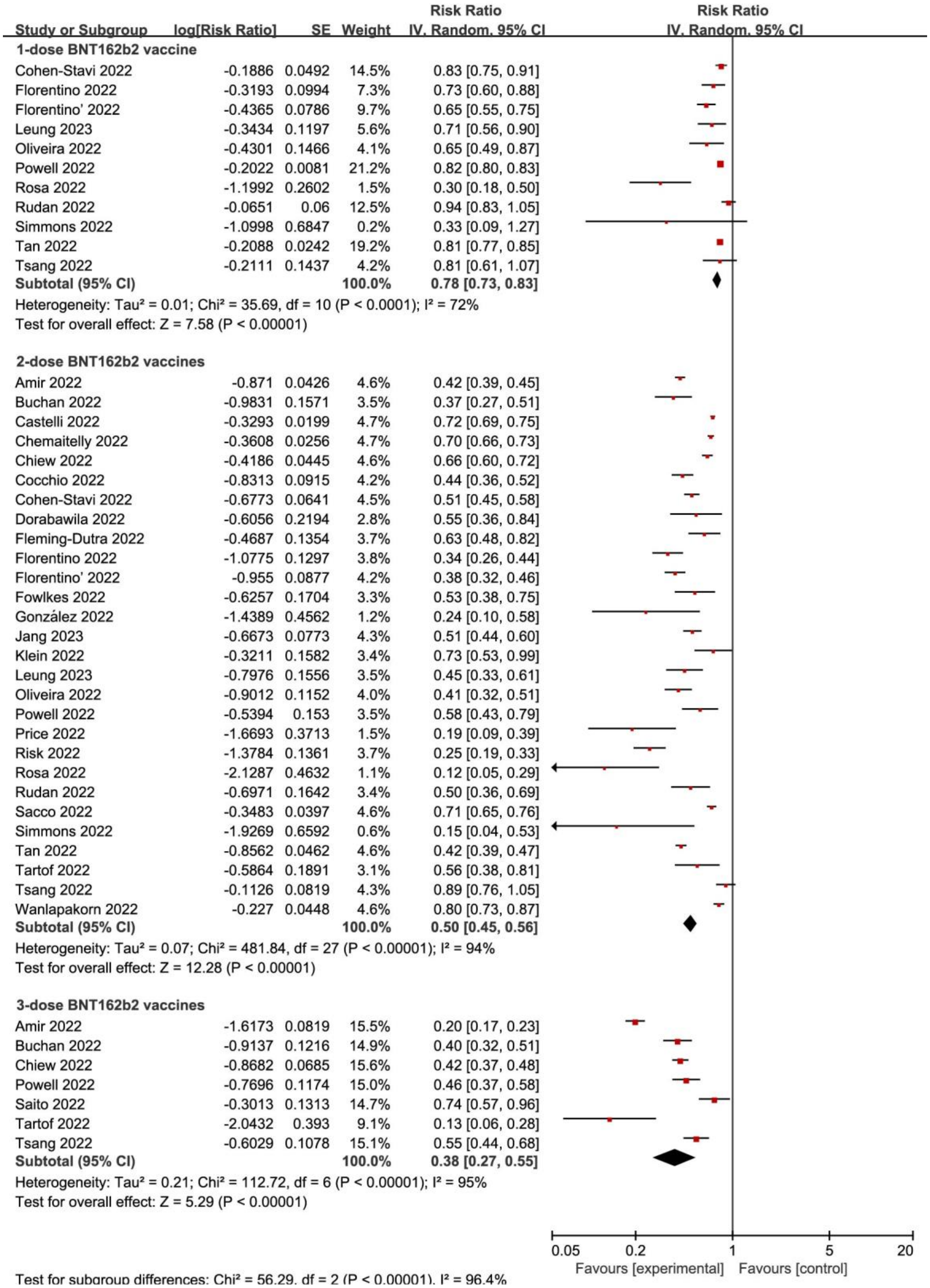

Supplemental Figure 6. Forest plot for vaccine effectiveness of different vaccines doses for the BNT162b2 vaccine on preventing Omicron infections. The red square symbolizes the point estimate for each study, with its size proportional to the study's weight relative to the summary estimate. The black diamond symbol represents the overall effect estimate derived from the meta-analysis. A random effects model was employed when I ²exceeded 50%.

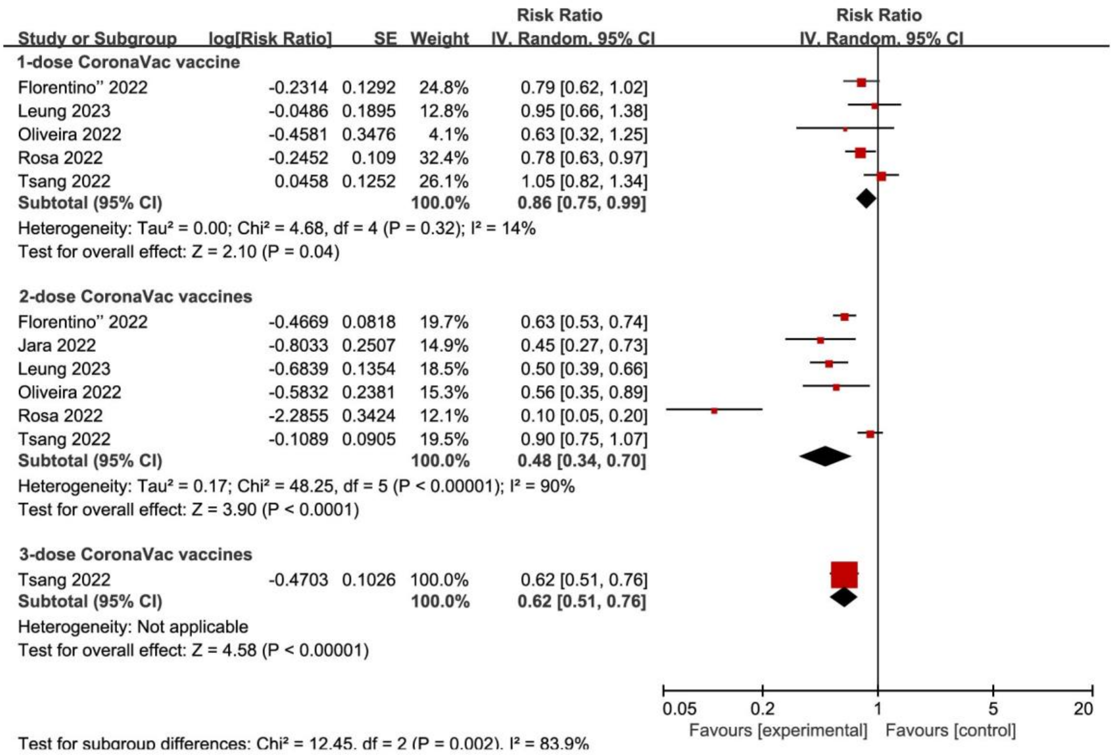

Supplemental Figure 7. Forest plot for vaccine effectiveness of different vaccines doses for the CoronaVac vaccine on preventing Omicron infections. The red square symbolizes the point estimate for each study, with its size proportional to the study's weight relative to the summary estimate. The black diamond symbol represents the overall effect estimate derived from the meta-analysis. A random effects model was employed when I^2 exceeded 50%.

Appendix 3: Supplementary Table

Supplemental Table 1. Characteristics of included studies.

**Name**	**Country**	**Study design**	**Age**	**Sample size (cases/total)**	**Vaccination type**	**Time**	**Main outcome**
Amir 2022	Israel	Cohort study	5-15 years	Not provided /1158289	2-dose BNT162b2 mRNA vaccine<br><br>3-dose BNT162b2 mRNA vaccine	14-35 days<br><br>14-60 days	In 5-10 years, the 2-dose decreased confirmed infection rates by 2.3 times (2.0-2.5) lower.<br><br>In 12-15 years, the 3-dose decreased confirmed infection rates by 3.3 times (2.8-4.0).
Tsang 2022	China	Cohort study	5-17 years	670/886	1-dose BNT162b2 mRNA vaccine<br><br>1-dose CoronaVac vaccines<br><br>2-dose BNT162b2 mRNA vaccine<br><br>2-dose CoronaVac vaccines	<3 months<br><br>≥3 months	COVID-19 Omicron infection for BNT162b2.<br><br>1-dose VE=28.6% (-33.9%-61.9%)<br><br>2-dose VE (≥3 months) =34.7% (-16.3%-63.3%)<br><br>2-dose VE (<3 months) = -15.1% (-292.2%-66.2%)<br><br>COVID-19 Omicron infection for CoronaVac.<br><br>1-dose VE=14.9% (-46.3%-50.5%)<br><br>2-dose VE (<3 months) =55.0% (-45.9%-86.1%)<br><br>COVID-19 Omicron symptomatic infection

Name	Country	Study design	Age	Sample size (cases/total)	Vaccination type	Time	Main outcome
							for BNT162b2.<br><br>1-dose VE=39.0% (-41.8%-73.8%)<br><br>2-dose VE (≥3 months) =54.5% (-5.3%-80.3%)<br><br>2-dose VE (<3 months) =-21.2% (-438.2%-72.7%)<br><br>COVID-19 Omicron symptomatic infection for CoronaVac.<br><br>1-dose VE=8.0% (-79.0%-52.7%)<br><br>2-dose VE (<3 months) = 28.6% (-139.3%-78.7%)
Saito 2022	Japan	Case-control study	13-18 years	1128/1558	3-dose BNT162b2 mRNA vaccine	<2 months	COVID-19 Omicron infection for BNT162b2<br>3-dose VE= 86.4% (57.2%-95.7%)
Sacco 2022	Italy	Case-control study	5-11 years	1197421 /2965918	1-dose BNT162b2 mRNA vaccine<br><br>2-dose BNT162b2 mRNA vaccine	<3 months	COVID-19 Omicron infection for BNT162b2 mRNA<br><br>1-dose VE= 27.4% (26.4%-28.4%)<br><br>2-dose VE= 29.4% (28.5%-30.2%)

**Name**	**Country**	**Study design**	**Age**	**Sample size (cases/total)**	**Vaccination type**	**Time**	**Main outcome**
							COVID-19 Omicron severe infection for BNT162b2 mRNA<br><br>1-dose VE= 38.1% (20.9%-51.5%)<br><br>2-dose VE= 41.1% (22.2%-55.4%)
Rudan 2022	Scotland	Cohort study	12-17 years	12-15 years：84300/237681<br><br>16-17 years：<br><br>27173/112609	2-dose BNT162b2 mRNA vaccine	<3 months<br><br>⩾3 months	COVID-19 Omicron symptomatic infection for BNT162b2 in 12-15 years.<br><br>1-dose VE (0-1week) =14.2% (-10.3%-33.2%)<br><br>1-dose VE (2-5weeks) =30.2% (18.4%-40.3%)<br><br>1-dose VE (6-9week) =21.8% (11.5%-30.8%)<br><br>1-dose VE (10-13weeks) =16.9% (8.7%-24.4%)<br><br>1-dose VE (14-17week) =9.5% (-3.6%-20.9%)<br><br>1-dose VE (＞18weeks) =5.4% (-13.4%-21.0%)<br><br>2-dose VE (0-1week) =46.9% (37%-55.3%)<br><br>2-dose VE (2-5weeks) =81.2% (77.7%-

Name	Country	Study design	Age	Sample size (cases/total)	Vaccination type	Time	Main outcome
							84.2%)<br><br>2-dose VE (6-9week) =68.5% (63.4%-72.9%)<br><br>2-dose VE (10-13weeks) =43.3% (30.0%-54.2%)<br><br>2-dose VE (＞14week) =48.7% (22.0%-66.3%)<br><br>COVID-19 Omicron symptomatic infection for BNT162b2 in 16-17 years.<br><br>1-dose VE (0-1week) = -18.4% (-89.3%-26.0%)<br><br>1-dose VE (2-5weeks) =22.8% (-6.4%-44.0%)<br><br>1-dose VE (6-9week) =11.9% (-16.1%-33.1%)<br><br>1-dose VE (10-13weeks) = -22.4% (-52.3%-1.6%)<br><br>1-dose VE (14-17week) = -24.2% (-46.5%- -5.3%)<br><br>1-dose (＞18weeks) VE= -24.7% (-46.7%- -

**Name**	**Country**	**Study design**	**Age**	**Sample size (cases/total)**	**Vaccination type**	**Time**	**Main outcome**
							6.0%)<br><br>2-dose VE (0-1week) =34.0% (13.2%-49.9%)<br><br>2-dose VE (2-5weeks) =65.5% (56.0%-73.0%)<br><br>2-dose VE (6-9week) =43.4% (26.9%-56.2%)<br><br>2-dose VE (10-13weeks) =8.9% (-19.1%-30.3%)<br><br>2-dose VE (＞14week) =1.2% (-49.3%-34.6%)
Rosa 2022	Hongkong, China	Case-control study	3-18 years	3-11 years: 143/1099<br><br>12-18 years: 306/455	1-dose BNT162b2 mRNA vaccine<br><br>1-dose CoronaVac vaccines<br><br>2-dose BNT162b2 mRNA vaccine<br><br>2-dose CoronaVac vaccines	Not provided	COVID-19 Omicron hospitalization for BNT162b2 in 3-11 years.<br><br>1-dose VE= 65.6% (38.2%-82.55)<br><br>COVID-19 Omicron hospitalization for CoronaVac in 3-11 years.<br><br>1-dose VE= 13.5% (-14.0%-34.6%)<br><br>2-dose VE= 86.2% (65.8%-95.9%)<br><br>COVID-19 Omicron hospitalization for BNT162b2 in 12-18 years.

Name	Country	Study design	Age	Sample size (cases/total)	Vaccination type	Time	Main outcome
							1-dose VE=60.2% (47.0%-70.2%)<br><br>2-dose VE=82.3% (76.9%-86.4%)<br><br>COVID-19 Omicron hospitalization for CoronaVac in 12-18 years.<br><br>1-dose VE= 31.0% (-9.34%-58.3%)<br><br>2-dose VE= 90.7% (79.2%-96.8%)<br><br>COVID-19 Omicron severe infection for BNT162b2 in 3-18 years.<br><br>1-dose VE= 84.6% (69.7%-93.2%)<br><br>2-dose VE= 93.1% (86.4%-97.0%)<br><br>COVID-19 Omicron severe infection for CoronaVac in 3-18 years.<br><br>1-dose VE= 33.7% (-4.6%-58.9%)<br><br>2-dose VE= 95.8% (80.7%-99.8%)
Risk 2022	American	Cohort study	12-17 years	2099/4332	2-dose BNT162b2 mRNA vaccine	<3 months<br><br>3-6 months	COVID-19 Omicron infection for 2-dose BNT162b2 mRNA.

**Name**	**Country**	**Study design**	**Age**	**Sample size (cases/total)**	**Vaccination type**	**Time**	**Main outcome**
						>6 months	VE (0-3month) = 54.5% (17.8%-76.9%)<br><br>VE (3-6month) = -25.0% (-68.8%-8.1%)<br><br>VE (＞6month) = 4.2% (-20.2%-23.6%)
Price 2022	America n	Case-control study	12-18 years	918/2275	2-dose BNT162b2 mRNA vaccine	2-22 weeks<br><br>23-24 weeks	COVID-19 Omicron hospitalization for 2-dose BNT162b2 in 12-18 years.<br><br>VE (2-22weeks) =43% (-1%-68%)<br><br>VE (23-24weeks) =38% (-3%-62%)<br><br>VE (no time limit) =40% (9%-60%)<br><br>COVID-19 Omicron hospitalization for 2-dose BNT162b2 in 5-11 years.<br><br>VE (no time limit) =68% (42%-82%)<br><br>COVID-19 Omicron severe infection for BNT162b2 in 12-18 years.<br><br>VE (no time limit) =79% (51%-91%)
Powell 2022	England	Case-control study	12-17 years	458122/ 1161704	1-dose BNT162b2 mRNA vaccine<br><br>2-dose BNT162b2	0–1 week<br><br>2–14 weeks	COVID-19 Omicron symptomatic infection for 1-dose BNT162b2.<br><br>VE (0-1week) = 15.2% (9.9%-20.1%)

Name	Country	Study design	Age	Sample size (cases/total)	Vaccination type	Time	Main outcome
					mRNA vaccine<br><br>3-dose BNT162b2 mRNA vaccine	15–24 weeks<br><br>25–39 weeks<br><br>⩾40 weeks	VE (2-14weeks) = 18.8% (17.2%-20.3%)<br><br>VE (15-24week) = 17.9% (14.9%-20.7%)<br><br>VE (25-39weeks) = 12.8% (-1.6%-25.1%)<br><br>VE (⩾40 weeks) = 14.7% (-19.9%-55.4%)<br><br>COVID-19 Omicron symptomatic infection for 2-dose BNT162b2.<br><br>VE (0-1week) = 52.2% (50.4%-53.9%)<br><br>VE (2-14weeks) = 64.5% (63.6%-65.4%)<br><br>VE (15-24week) = 29.8% (24.9%-34.2%)<br><br>VE (25-39weeks) = 19.4% (11.7%-26.4%)<br><br>VE (⩾40 weeks) = 25.7% (-4.2%-47.0%)<br><br>COVID-19 Omicron symptomatic infection for 3-dose BNT162b2.<br><br>VE (0-1week) = 55.1% (50.7%-59.1%)<br><br>VE (2-14weeks) = 62.9% (60.5%-65.1%)<br><br>VE (15-24week) = 33.6% (14.6%-48.3%)

**Name**	**Country**	**Study design**	**Age**	**Sample size (cases/total)**	**Vaccination type**	**Time**	**Main outcome**
Wanlapakorn<br><br>2022	Thailand	Cohort study	5-11 years	85/166	1-dose CoronaVac vaccine +1-dose BNT162b2 vaccine<br><br>2-dose BNT162b2 mRNA vaccine<br><br>2-dose BBIBP-CorV vaccine + 1-dose BNT162b2 vaccine	＞1 month	Neutralizing activity against Omicron infection for 1-dose CoronaVac vaccine +1-dose BNT162b2 vaccine.<br><br>Median (IQR)% =50.8(45.2-66.4)<br><br>Neutralizing activity against Omicron infection for 2-dose BNT162b2 mRNA vaccine.<br><br>Median (IQR)%=79.8(72.5-87.0)<br><br>Neutralizing activity against Omicron infection for 2-dose BBIBP-CorV vaccine + 1-dose BNT162b2 vaccine.<br><br>Median (IQR)%=82.2(71.7-87.5)
Florentino 2022	Scotland	Case-control study	12-17 years	26177/45771	1-dose BNT162b2 mRNA vaccine<br><br>2-dose BNT162b2 mRNA vaccine	0-6 days<br><br>7-13 days<br><br>⩾14 days<br><br>0-13 days<br><br>14-27 days<br><br>28-41 days	COVID-19 Omicron symptomatic infection for 1-dose BNT162b2.<br><br>VE (0-6 days) = 10.1% (-14.2%-29.1%)<br><br>VE (7-13 days) = 42.5% (29.4%-53.3%)<br><br>VE (≥14 days) =25.1% (21.3%-28.7%)<br><br>COVID-19 Omicron symptomatic infection for 2-dose BNT162b2.

Name	Country	Study design	Age	Sample size (cases/total)	Vaccination type	Time	Main outcome
						42-55 days<br>56-69 days<br>70-83 days<br>84-97 days<br>⩾98 days	VE (0-13 days) = 61.0% (56.9%-64.8%)<br>VE (14-27 days) = 82.6% (80.6%-84.5%)<br>VE (28-41 days) = 77.4% (74.7%-79.8%)<br>VE (42-55 days) = 69.6% (66.3%-72.6%)<br>VE (56-69 days) = 65.4% (61.9%-68.7%)<br>VE (70-83 days) = 58.0% (52.9%-62.6%)<br>VE (84-97 days) = 45.3% (37.2%-52.4%)<br>VE (⩾98 days) = 50.6% (42.7%-57.4%)
Florentino' 2022	Brazil	Case-control study	12-17 years	150291/355066	1-dose BNT162b2 mRNA vaccine<br>2-dose BNT162b2 mRNA vaccine	0-6 days<br>7-13 days<br>⩾14 days<br>0-13 days<br>14-27 days<br>28-41 days	COVID-19 Omicron symptomatic infection for 1-dose BNT162b2.<br>VE (0-6 days) = 37.5% (28.5%-45.3%)<br>VE (7-13 days) = 19.7% (10.6%-27.9%)<br>VE (≥14 days) = 28.0% (26.3%-29.7%)<br>COVID-19 Omicron symptomatic infection for 2-dose BNT162b2.

**Name**	**Country**	**Study design**	**Age**	**Sample size (cases/total)**	**Vaccination type**	**Time**	**Main outcome**
						42-55 days<br>56-69 days<br>70-83 days<br>84-97 days<br>⩾98 days	VE (0-13 days) = 58.7% (56.4%-61.0%)<br>VE (14-27 days) = 64.7% (63.0%-66.3%)<br>VE (28-41 days) = 53.0% (51.3%-54.7%)<br>VE (42-55 days) = 40.6% (38.8%-42.4%)<br>VE (56-69 days) = 32.0% (30.0%-33.9%)<br>VE (70-83 days) = 25.3% (22.9%-27.6%)<br>VE (84-97 days) = 17.0% (13.8%-20.0%)<br>VE (⩾98 days) = 5.9% (2.2%-9.4%)<br>COVID-19 Omicron severe infection for 1-dose BNT162b2.<br>VE (0-6 days) = 20.6% (-152.2%-75.0%)<br>VE (7-13 days) = 62.4% (-22.2%-88.5%)<br>VE (≥14 days) =56.3% (45.9%-64.6%)<br>COVID-19 Omicron severe infection for 1-dose BNT162b2.<br>VE (0-13 days) = 65.0% (37.2%-80.5%)<br>VE (14-27 days) =75.6% (58.1%-85.8%)

Name	Country	Study design	Age	Sample size (cases/total)	Vaccination type	Time	Main outcome
							VE (28-41 days) = 82.8% (72.1%-89.4%)<br><br>VE (42-55 days) = 84.2% (76.3%-89.5%)<br><br>VE (56-69 days) = 83.7% (76.0%-88.9%)<br><br>VE (70-83 days) = 82.0% (72.6%-88.2%)<br><br>VE (84-97 days) = 86.4% (75.2%-92.6%)<br><br>VE (⩾98 days) =82.7% (68.8%-90.4%)
Florentino '' 2022	Brazil	Case-control study	6-11 years	89595/197958	1-dose CoronaVac vaccine<br><br>2-dose CoronaVac vaccine	0-13 days<br><br>⩾14 days	COVID-19 Omicron symptomatic infection for 1-dose CoronaVac vaccine.<br><br>VE (0-13 days) = -9.0% (-13.1%--4.9%)<br><br>VE (≥14 days) = 21.2% (18.6%-23.8%)<br><br>COVID-19 Omicron symptomatic infection for 2-dose CoronaVac vaccine.<br><br>VE (0-13 days) = 30.8% (24.2%-36.8%)<br><br>VE (≥14 days) = 39.8% (33.7%-45.4%)<br><br>COVID-19 Omicron severe infection for 1-dose CoronaVac vaccine.

**Name**	**Country**	**Study design**	**Age**	**Sample size (cases/total)**	**Vaccination type**	**Time**	**Main outcome**
							VE (0-13 days) = 27.0% (-5.2%-51.1%)<br><br>VE (≥14 days) = 47.1% (26.6%-62.7%)<br><br>COVID-19 Omicron severe infection for 2-dose CoronaVac vaccine.<br><br>VE (0-13 days) = 82.4% (44.2%-97.1%)<br><br>VE (≥14 days) = 59.2% (11.3%-84.5%)
Nordström 2022	Sweden	Cohort study	11-19 years	501945/659924	2-dose COVID-19 mRNA vaccine	No time limit	COVID-19 Omicron infection for 2-dose COVID-19 mRNA vaccine.<br><br>VE= -2% (-4%—0%)<br><br>COVID-19 hospitalisation for 2-dose COVID-19 mRNA vaccine.<br><br>VE= 75% (54%-86%)
Chiew 2022	Singapore	Cohort study	12-17 years	245028/249763	2-dose BNT162b2 mRNA vaccine<br><br>3-dose BNT162b2 mRNA vaccine	1-7 days<br><br>8-59 days<br><br>60-89 days<br><br>90-119 days<br><br>120-149 days	COVID-19 Omicron infection for BNT162b2.<br><br>2-dose VE (no time limit) = 25% (21%-29%)<br><br>3-dose VE (no time limit) = 56% (53%-58%)<br><br>COVID-19 Omicron hospitalization for BNT162b2.

Name	Country	Study design	Age	Sample size (cases/total)	Vaccination type	Time	Main outcome
						150-179 days<br>180-209 days<br>210-239 days<br>⩾240 days<br>1-7 days<br>8-14 days<br>14-29 days<br>30-59 days<br>⩾60 days	2-dose VE (no time limit) = 75% (56%-86%)<br>3-dose VE (no time limit) =94% (86%-97%)<br>COVID-19 Omicron infection for 2-dose BNT162b2.<br>VE (1-7 days) = 48% (33%-60%)<br>VE (8-59 days) = 38% (33%-43%)<br>VE (60-89 days) = 33% (27%-38%)<br>VE (90-119 days) = 26% (21%-31%)<br>VE (120-149 days) = 19% (14%-25%)<br>VE (150-179 days) = 16% (10%-22%)<br>VE (180-209 days) = 18% (14%-23%)<br>VE (210-239 days) = 21% (16%-25%)<br>VE (⩾240 days) = 41% (35%-46%)<br>COVID-19 Omicron hospitalization for 2-dose BNT162b2.<br>VE (8-59 days) = 76% (27%-92%)

Name	Country	Study design	Age	Sample size (cases/total)	Vaccination type	Time	Main outcome
							VE (60-89 days) = 90% (51%-98%)<br><br>VE (90-119 days) = 65% (15%-86%)<br><br>VE (120-149 days) = 70% (26%-89%)<br><br>VE (150-179 days) = 60% (19%-80%)<br><br>VE (180-209 days) = 83% (65%-91%)<br><br>VE (210-239 days) = 78% (55%-90%)<br><br>COVID-19 Omicron infection for 3-dose BNT162b2.<br><br>VE (1-7 days) = 47% (43%-49%)<br><br>VE (8-14 days) = 60% (57%-63%)<br><br>VE (14-29 days) = 61% (58%-63%)<br><br>VE (30-59 days) = 53% (49%-55%)<br><br>VE (⩾60 days) = 41% (35%-45%)<br><br>COVID-19 Omicron hospitalization for 3-dose BNT162b2.<br><br>VE (1-7 days) = 87% (67%-95%)<br><br>VE (8-14 days) = 97% (83%-100%)

Name	Country	Study design	Age	Sample size (cases/total)	Vaccination type	Time	Main outcome
							VE (14-29 days) = 98% (89%-100%)<br><br>VE (30-59 days) = 89% (65%-96%)<br><br>VE (⩾60 days) = 85% (15%-98%)
Tan 2022	Singapor e	Cohort study	5-11 years	203893/255936	1-dose BNT162b2 mRNA vaccine<br><br>2-dose BNT162b2 mRNA vaccine	1-6 days<br><br>7-14 days<br><br>15-29 days<br><br>⩾30 days<br><br>30-59 days<br><br>⩾60 days	COVID-19 Omicron infection for 1-dose BNT162b2 in 5-11 years.<br><br>VE (no time limit) = 13.6% (11.7%-15.5%)<br><br>VE (1-6 days) = 20.9% (16.6%-25.0%)<br><br>VE (7-14 days) = -1.3% (-5.1%-2.4%)<br><br>VE (15-29 days) = 2.0% (-1.0%-4.8%)<br><br>VE (⩾30 days) =21.9% (18.1%-25.6%)<br><br>COVID-19 Omicron hospitalizations for 1-dose BNT162b2 in 5-11 years.<br><br>VE (no time limit) = 42.3% (24.9%-55.7%)<br><br>VE (1-6 days) = 34.5% (-18.5%-63.8%)<br><br>VE (7-14 days) =34.5% (-18.5%-63.8%)

Name	Country	Study design	Age	Sample size (cases/total)	Vaccination type	Time	Main outcome
							VE (15-29 days) = 44.2% (18.9%-61.6%)<br>VE (⩾30 days) = 51.6% (7.1%-74.8%)<br>COVID-19 Omicron infection for 2-dose BNT162b2 in 5-11 years.<br>VE (no time limit) = 36.8% (35.3%-38.2%)<br>VE (1-6 days) = 35.7% (33.0%-38.2%)<br>VE (7-14 days) = 48.8% (46.9%-50.8%)<br>VE (15-29 days) = 37.6% (35.7%-39.3%)<br>VE (30-59 days) = 28.5% (26.3%-30.7%)<br>VE (⩾60 days) = 28.5% (26.3%-30.7%)<br>COVID-19 Omicron hospitalizations for 2-dose BNT162b2 in 5-11 years.<br>VE (no time limit) = 82.7% (74.8%-88.2%)<br>VE (1-6 days) = 64.7% (37.3%-80.2%)<br>VE (7-14 days) = 87.8% (72.2%-94.7%)<br>VE (15-29 days) = 84.5% (72.7%-91.2%)

**Name**	**Country**	**Study design**	**Age**	**Sample size (cases/total)**	**Vaccination type**	**Time**	**Main outcome**
							VE (30-59 days) = 80.4% (67.0%—88.4%)
Tartof 2022	Ameriaca	Cohort study	12-17 years	2016/3168	2-dose BNT162b2 mRNA vaccine	<2 month<br><br>2-3 months<br><br>4-5 months<br><br>⩾6 months	COVID-19 Omicron infection for 2-dose BNT162b2.<br><br>VE (<2 month) =73% (54%-84%)<br><br>VE (2-3 months) = 38% (14%-56%)<br><br>VE (4-5 months) = 45% (28%-57%)<br><br>VE (⩾6 months) = 16% (-7%-34%)
Cocchio 2022	Italian	Cross-sectional study	5-17 years	225983/585546	2-dose BNT162b2 mRNA vaccine<br><br>2-dose mRNA-1273 vaccine<br><br>1-dose mRNA-1273 + 1-dose BNT162b2	0–6 d<br><br>7–13 d<br><br>14–34 d<br><br>35–69 d<br><br>＞70 d	COVID-19 Omicron infection for 2-dose BNT162b2 in 5-11 years.<br><br>VE (no time limit) = 35% (34%–37%)<br><br>VE (0-6 days) = 72% (69%–74%)<br><br>VE (7-13 days) =70% (67% ‑ 72%)<br><br>VE (14-34 days) = 53% (51%–55%)<br><br>VE (35-69 days) = 22% (19%–24%)<br><br>VE (⩾70 days) = 23% (20%–26%)

**Name**	**Country**	**Study design**	**Age**	**Sample size (cases/total)**	**Vaccination type**	**Time**	**Main outcome**
							COVID-19 Omicron infection for 2-dose BNT162b2 in 12-17 years.<br><br>VE (no time limit) = 17% (15%–20%)<br><br>VE (0-6 days) = 81% (76%–85%)<br><br>VE (7-13 days) = 83% (79%–86%)<br><br>VE (14-34 days) = 59% (55%–62%)<br><br>VE (35-69 days) = 23% (19%–27%)<br><br>VE (⩾70 days) = 8% (5%–11%)<br><br>COVID-19 Omicron infection for 2-dose mRNA-1273 in 12-17 years.<br><br>VE (no time limit) = 30% (26%–33%)<br><br>VE (0-6 days) = 88% (81%–92%)<br><br>VE (7-13 days) = 78% (69%–84%)<br><br>VE (14-34 days) = 55% (49%–61%)<br><br>VE (35-69 days) = 29% (23%–35%)<br><br>VE (⩾70 days) = 20% (15%–24%)<br><br>COVID-19 Omicron infection for 3-dose

Name	Country	Study design	Age	Sample size (cases/total)	Vaccination type	Time	Main outcome
							BNT162b2 in 12-17 years.<br><br>VE (no time limit) = 38% (36%–40%)<br><br>VE (0-6 days) = 79% (77%–81%)<br><br>VE (7-13 days) = 80% (78%–82%)<br><br>VE (14-34 days) = 72% (70%–73%)<br><br>VE (35-69 days) = 30% (27%–33%)<br><br>COVID-19 Omicron infection for 1-dose mRNA-1273 + 1-dose BNT162b2 in 12-17 years.<br><br>VE (no time limit) = 53% (50%–56%)<br><br>VE (0-6 days) = 82% (77%–86%)<br><br>VE (7-13 days) = 80% (75%–84%)<br><br>VE (14-34 days) = 71% (66%–74%)<br><br>VE (35-69 days) = 32% (26%–38%)
Cohen-Stavi	Israel	Cohort study	5-11 years	94728/189456	1-dose BNT162b2 mRNA vaccine	7-21 days	COVID-19 Omicron infection for BNT162b2 in 5-11 years.

**Name**	**Country**	**Study design**	**Age**	**Sample size (cases/total)**	**Vaccination type**	**Time**	**Main outcome**
2022					2-dose BNT162b2 mRNA vaccine	14-27 days	1-dose VE (14-27 days) = 17% (7%-25%)<br><br>2-dose VE (7-21 days) = 51% (39%-61%)<br><br>COVID-19 Omicron symptomatic infection for BNT162b2 in 5-11 years.<br><br>1-dose VE (14-27 days) = 18% (-2%-34%)<br><br>2-dose VE (7-21 days) = 48% (29%-63%)<br><br>COVID-19 Omicron infection for 2-dose BNT162b2 in 5-6 years.<br><br>VE (7-21 days) = 68% (43%-84%)<br><br>COVID-19 Omicron symptomatic infection for 2-dose BNT162b2 in 5-6 years.<br><br>VE (7-21 days) = 69% (30%-91%)<br><br>COVID-19 Omicron infection for 2-dose BNT162b2 in 7-9 years.<br><br>VE (7-21 days) = 56% (41%-68%)<br><br>COVID-19 Omicron symptomatic infection for 2-dose BNT162b2 in 7-9 years.<br><br>VE (7-21 days) = 49% (6%-76%)

Name	Country	Study design	Age	Sample size (cases/total)	Vaccination type	Time	Main outcome
							COVID-19 Omicron infection for 2-dose BNT162b2 in 10-11 years.<br><br>VE (7-21 days) = 38% (18%-53%)<br><br>COVID-19 Omicron symptomatic infection for 2-dose BNT162b2 in 10-11 years.<br><br>VE (7-21 days) = 36% (0%-61%)
Fleming-Dutra 2022	America	Case-control study	5-15 years	53272 /121952	2-dose BNT162b2 mRNA vaccine<br><br>3-dose BNT162b2 mRNA vaccine	0-2 months<br><br>⩾month 3	COVID-19 Omicron symptomatic infection for 2-dose BNT162b2 in 5-11 years.<br><br>VE (0-2 months) = 60.1% (54.7%-64.8%)<br><br>VE (＞2 months) = 28.9% (24.5%-33.1%)<br><br>COVID-19 Omicron symptomatic infection for 2-dose BNT162b2 in 12-15 years.<br><br>VE (0-2 months) = 59.5% (44.3%-70.6%)<br><br>VE (＞2 months) =16.6% (8.1%-24.3%)<br><br>VE (⩾3 months) = 9.6% (−0.1%-18.3%)<br><br>COVID-19 Omicron symptomatic infection for 3-dose BNT162b2 in 12-15 years.

Name	Country	Study design	Age	Sample size (cases/total)	Vaccination type	Time	Main outcome
							VE (2-6.5 weeks) =71.1% (65.5%-75.7%)
Dorabawila 2022	America	Cohort study	5-17 years	1217886/2423585	2-dose BNT162b2 mRNA vaccine	⩽13 days<br><br>28-34 days	COVID-19 Omicron infection for 2-dose BNT162b2 in 5-11 years.<br><br>VE (no time limit) = 12% (6%-16%)<br><br>COVID-19 Omicron infection for 2-dose BNT162b2 in 12-17 years.<br><br>VE (no time limit) = 51% (48%-54%)<br><br>COVID-19 Omicron hospitalizations for 2-dose BNT162b2 in 5-11 years.<br><br>VE (no time limit) = 48% (-12%-75%)<br><br>COVID-19 Omicron hospitalizations for 2-dose BNT162b2 in 12-17 years.<br><br>VE (no time limit) = 73% (53%-87%)<br><br>COVID-19 Omicron symptomatic infection for 2-dose BNT162b2 in 5-11 years.<br><br>VE (⩽13 days) = 65% (62%-68%)<br><br>VE (28-34 days) = 12% (8%-16%)<br><br>COVID-19 Omicron symptomatic infection

**Name**	**Country**	**Study design**	**Age**	**Sample size (cases/total)**	**Vaccination type**	**Time**	**Main outcome**
							for 2-dose BNT162b2 in 12-17 years.<br><br>VE (⩽13 days) = 76% (71%-81%)<br><br>VE (28-34 days) = 56% (48%-63%)
Buchan 2022	Canada	Case-control study	12-17 years	27556/29855	2-dose BNT162b2 mRNA vaccine<br><br>3-dose BNT162b2 mRNA vaccine	1-6 days<br><br>7-59 days<br><br>60-119 days<br><br>120-179 days<br><br>⩾180 days	COVID-19 Omicron symptomatic infection for 2-dose BNT162b2.<br><br>VE (7-59 days) = 51% (38%-61%)<br><br>VE (60-119 days) = 31% (20%-41%)<br><br>VE (120-179 days) = 29% (19%-38%)<br><br>VE (⩾180 days) =29% (17%-38%)<br><br>COVID-19 Omicron symptomatic infection for 3-dose BNT162b2.<br><br>VE (1-6 days) = 56% (34%-70%)<br><br>VE (⩾7 days) = 62% (49%-72%)<br><br>COVID-19 Omicron severe infection for 2-dose BNT162b2.<br><br>VE (7-59 days) = 76% (-10%-95%)

Name	Country	Study design	Age	Sample size (cases/total)	Vaccination type	Time	Main outcome
							VE (60-119 days) = 83% (55%-93%)<br><br>VE (120-179 days) = 82% (64%-91%)<br><br>VE (⩾180 days) =88% (77%-94%)<br><br>COVID-19 Omicron severe infection for 3-dose BNT162b2.<br><br>VE (⩾7 days) =85% (74%-91%)
Simmons 2022	Canada	Case-control study	12-17 years	Not provided /673	1-dose BNT162b2 mRNA vaccine<br><br>2-dose BNT162b2 mRNA vaccine	No time limit	COVID-19 Omicron hospitalizations for 2-dose BNT162b2.<br><br>OR=0.67 (0.08-6.80)<br><br>COVID-19 Omicron hospitalizations for 3-dose BNT162b2.<br><br>OR= 0.20 (0.08-0.59)
Fowlkes 2022	America	Cohort study	5-15 years	978/1364	2-dose BNT162b2 mRNA vaccine	14-82 days<br><br>⩾14 days<br><br>14-149 days<br><br>⩾150 days	COVID-19 Omicron symptomatic infection for 2-dose BNT162b2 in 5-11 years.<br><br>VE (14-82 days) = 31.0% (9.0%-48.0%)<br><br>COVID-19 Omicron symptomatic infection for 2-dose BNT162b2 in 12-15 years.

Name	Country	Study design	Age	Sample size (cases/total)	Vaccination type	Time	Main outcome
							VE (⩾14 days) = 59.0% (24.0%-78.0%)<br>VE (14-149 days) = 59.0% (22.0%-79.0%)<br>VE (⩾150 days) =62.0% (-28.0%-89.0%)
Klein 2022	America	Case-control study	5-17 years	2577/12214	2-dose BNT162b2 mRNA vaccine<br>3-dose BNT162b2 mRNA vaccine	14-67 days<br>14-149 days<br>⩾150 days	COVID-19 Omicron infection for 2-dose BNT162b2 in 5-11 years.<br>VE (14-67 days) = 51.0% (30.0%-65%)<br>COVID-19 Omicron infection for 2-dose BNT162b2 in 12-15 years.<br>VE (14-149 days) = 45.0% (30.0%-57%)<br>VE (⩾150 days) = -2.0% (-25.0%-17.0%)<br>COVID-19 Omicron infection for 2-dose BNT162b2 in 16-17 years.<br>VE (14-149 days) = 34.0% (8.0%-53.0%)<br>VE (⩾150 days) = -3.0% (-30.0%-18.0%)<br>COVID-19 Omicron infection for 3-dose BNT162b2 in 16-17 years.

**Name**	**Country**	**Study design**	**Age**	**Sample size (cases/total)**	**Vaccination type**	**Time**	**Main outcome**
							VE (⩾7 days) = 81.0% (59.0%-91.0%)
Castelli 2022	Argentina	Case-control study	3-17 years	188907/249112	2-dose BNT162b2 mRNA vaccine<br><br>2-dose BBIBP-CorV vaccine<br><br>2-dose mRNA-1273 vaccine<br><br>1-dose mRNA-1273+ 1-dose BNT162b2<br><br>1-dose BNT162b2/mRNA-1273+1-dose BNT162b2/mRNA-1273	15-30 days<br><br>31-45 days<br><br>45-60 days<br><br>⩾60 days	COVID-19 Omicron infection for 2-dose vaccination in 3-17 years.<br><br>VE (no time limit) = 19.9% (18.0%-21.8%)<br><br>COVID-19 Omicron infection for 2-dose BBIBP-CorV vaccine in 3-11 years.<br><br>VE (no time limit) = 15.9% (13.2%-18.6%)<br><br>VE (15-30 days) = 37.6% (34.2%-40.8%)<br><br>VE (31-45 days) = 29.4% (26.2%-32.4%)<br><br>VE (45-60 days) = 17.6% (14.1%-20.9%)<br><br>VE (⩾60 days) = 2.0% (-1.8%-5.6%)<br><br>COVID-19 Omicron infection for 2-dose mRNA-1273 vaccine in 12-17 years.<br><br>VE (no time limit) =17.9% (14.0%-21.5%)<br><br>COVID-19 Omicron infection for 1-dose mRNA-1273+ 1-dose BNT162b2 in 12-17 years.<br><br>VE (no time limit) =31.5% (26.3%-36.4%)

Name	Country	Study design	Age	Sample size (cases/total)	Vaccination type	Time	Main outcome
							COVID-19 Omicron infection for 1-dose BNT162b2+ 1-dose mRNA-1273 in 12-17 years.<br><br>VE (no time limit) = 40.6% (29.4%-50.0%)<br><br>COVID-19 Omicron infection for 2-dose BNT162b2 in 12-17 years.<br><br>VE (no time limit) = 28.1% (25.2%-30.8%)<br><br>COVID-19 Omicron severe infection for 2-dose vaccination in 3-17 years.<br><br>VE (no time limit) = 88.1% (70.7%-95.2%)<br><br>COVID-19 Omicron severe infection for 2-dose BBIBP-CorV vaccine in 3-11 years.<br><br>VE (no time limit) =66.9% (6.4%-89.8%)<br><br>COVID-19 Omicron severe infection for 1-dose BNT162b2/mRNA-1273+1-dose BNT162b2/mRNA-1273 in 12-17 years.<br><br>VE (no time limit) =97.6% (81.0%-99.7%)
Wang	China	Case-control	0-17 years	126/376	1-dose vaccination	No time limit	COVID-19 Omicron symptomatic infection

**Name**	**Country**	**Study design**	**Age**	**Sample size (cases/total)**	**Vaccination type**	**Time**	**Main outcome**
2022		study			2-dose vaccination		for 1-dose vaccination in 0-17 years.<br><br>RR= 0.73 (0.47–1.13)<br><br>COVID-19 Omicron symptomatic infection for 2-dose vaccination in 0-17 years.<br><br>RR= 0.65 (0.53–0.79)<br><br>COVID-19 Omicron symptomatic infection for 1-dose vaccination in 3-17 years.<br><br>RR= 0.71 (0.57–0.88)<br><br>COVID-19 Omicron symptomatic infection for 2-dose vaccination in 0-17 years.<br><br>RR= 0.80 (0.51–1.24)
Leung 2023	Hongkong, China	Case-control study	3-18 years	757433/953400	1-dose CoronaVac vaccine<br><br>1-dose BNT162b2 mRNA vaccine<br><br>2-dose CoronaVac vaccine<br><br>2-dose BNT162b2 mRNA vaccine	Not provided	COVID-19 Omicron symptomatic infection for 1-dose CoronaVac vaccine in 3-11 years.<br><br>VE= -14.7% (-54.7%-14.6%)<br><br>COVID-19 Omicron symptomatic infection for 1-dose BNT162b2 mRNA in 3-11 years.<br><br>VE= 33.0% (3.0%-53.3%)<br><br>COVID-19 Omicron symptomatic infection

Name	Country	Study design	Age	Sample size (cases/total)	Vaccination type	Time	Main outcome
							for 2-dose CoronaVac vaccine in 3-11 years.<br><br>VE= 40.8% (12.8%-59.5%)<br><br>COVID-19 Omicron symptomatic infection for 1-dose CoronaVac vaccine in 12-18 years.<br><br>VE= 21.5% (-7.7%-42.7%)<br><br>COVID-19 Omicron symptomatic infection for 1-dose BNT162b2 mRNA in 12-18 years.<br><br>VE= 26.1% (-0.3%-45.6%)<br><br>COVID-19 Omicron symptomatic infection for 2-dose CoronaVac vaccine in 12-18 years.<br><br>VE= 55.0% (38.2%-67.2%)<br><br>COVID-19 Omicron symptomatic infection for 2-dose BNT162b2 mRNA in 12-18 years.<br><br>VE= 54.9% (38.9%-66.8%)
Chemaitelly 2022	Qatar	Cohort study	5-17 years	21888/113758	2-dose BNT162b2 mRNA vaccine	0-30 days<br><br>31-60 days	COVID-19 Omicron infection for 2-dose BNT162b2 mRNA in 5-11 years.<br><br>VE (no time limit) = 25.7% (10.0%-38.6%)

**Name**	**Country**	**Study design**	**Age**	**Sample size (cases/total)**	**Vaccination type**	**Time**	**Main outcome**
						61-90 days<br><br>⩾91 days	VE (0-30 days) = 49.6% (28.5%-64.5%)<br><br>VE (31-60 days) = 23.0% (-14.0%-48.1%)<br><br>VE (61-90 days) = 11.0% (−26.8%-37.5%)<br><br>VE (⩾91 days) = -9% (−30.0%-32.0%)<br><br>COVID-19 Omicron infection for 2-dose BNT162b2 mRNA in 15-17 years.<br><br>VE (no time limit) = 30.6% (26.9%-34.1%)<br><br>VE (⩾14 days) = 51.3% (34.9%-63.6%)<br><br>VE (14-59 days) = 41.1% (36.1%-45.3%)<br><br>VE (60-149 days) =24.2% (17.5%-30.4%)<br><br>VE (150-300 days) = −1.7% (−16.9%-11.5%)<br><br>COVID-19 Omicron infection for 2-dose BNT162b2 mRNA in 12-14 years.<br><br>VE (no time limit) = 35.6% (31.2%-39.6%)<br><br>COVID-19 Omicron infection for 2-dose BNT162b2 mRNA in 15-17 years.<br><br>VE (no time limit) = 20.9% (13.8%-27.4%)

Name	Country	Study design	Age	Sample size (cases/total)	Vaccination type	Time	Main outcome
							COVID-19 Omicron symptomatic infection for 2-dose BNT162b2 mRNA in 12-17 years.<br><br>VE (no time limit) = 43.6% (35.1%-50.9%)
Oliveira 2022	Brail	Case-control study	12-19 years	3705/8458	1-dose BNT162b2/ChAdOx1 nCoV-19/CoronaVac<br><br>2-dose BNT162b2/ChAdOx1 nCoV-19/CoronaVac<br><br>1-dose BNT162b2 mRNA vaccine<br><br>2-dose BNT162b2 mRNA vaccine<br><br>1-dose ChAdOx1 nCoV-19 vaccine<br><br>2-dose ChAdOx1 nCoV-19 vaccine<br><br>1-dose CoronaVac	14-30 days<br><br>31-60 days<br><br>61-90 days<br><br>>90 days	COVID-19 Omicron hospitalization for 1-dose BNT162b2/ChAdOx1 nCoV-19/CoronaVac in 12-19 years.<br><br>VE (no time limit) =39.9% (25.0%-50.7%)<br><br>COVID-19 Omicron hospitalization for 1-dose BNT162b2/ChAdOx1 nCoV-19/CoronaVac in 12-15 years.<br><br>VE (no time limit) = 49.0% (29.0%-63.0%)<br><br>COVID-19 Omicron hospitalization for 1-dose BNT162b2/ChAdOx1 nCoV-19/CoronaVac in 16-17 years.<br><br>VE (no time limit) = 17.0% (-26.0%-45.0%)<br><br>COVID-19 Omicron hospitalization for 1-dose BNT162b2/ChAdOx1 nCoV-19/CoronaVac in 18-19 years.<br><br>VE (no time limit) = 39.0% (11.0%-59.0%)

Name	Country	Study design	Age	Sample size (cases/total)	Vaccination type	Time	Main outcome
					vaccine<br><br>2-dose CoronaVac vaccine		COVID-19 Omicron hospitalization for 2-dose BNT162b2/ChAdOx1 nCoV-19/CoronaVac in 12-19 years.<br><br>VE (no time limit) =59.0% (49.0%-66.0%)<br><br>VE (14-30 days) = 75% (55.0%-86.0%)<br><br>VE (31-60 days) = 62.1% (46.9%-70.7%)<br><br>VE (61-90 days) = 57.9% (42.9%-69.7%)<br><br>VE (⩾90 days) = 54.0% (39.0%-68.0%)<br><br>COVID-19 Omicron hospitalization for 2-dose BNT162b2/ChAdOx1 nCoV-19/CoronaVac in 12-15 years.<br><br>VE (no time limit) = 63.0% (48.0%-74.0%)<br><br>COVID-19 Omicron hospitalization for 2-dose BNT162b2/ChAdOx1 nCoV-19/CoronaVac in 16-17 years.<br><br>VE (no time limit) = 46.0% (20.0%-54.0%)<br><br>COVID-19 Omicron hospitalization for 2-dose BNT162b2/ChAdOx1 nCoV-19/CoronaVac in 18-19 years.

Name	Country	Study design	Age	Sample size (cases/total)	Vaccination type	Time	Main outcome
							VE (no time limit) = 60.0% (45.0%-72.0%)<br><br>COVID-19 Omicron hospitalization for 1-dose BNT162b2 in 12-18 years.<br><br>VE (no time limit) = 35.0% (16.0%-59.0%)<br><br>COVID-19 Omicron hospitalization for 2-dose BNT162b2 in 12-18 years.<br><br>VE (no time limit) = 60.0% (47.0%-69.0%)<br><br>COVID-19 Omicron hospitalization for 1-dose BNT162b2 in 18-19 years.<br><br>VE (no time limit) = 35.0% (-10.0%-58.0%)<br><br>COVID-19 Omicron hospitalization for 2-dose BNT162b2 in 18-19 years.<br><br>VE (no time limit) = 58.0% (35.0%-72.0%)<br><br>COVID-19 Omicron hospitalization for 1-dose ChAdOx1 nCoV-19 in 18-19 years.<br><br>VE (no time limit) =55.0% (15.0%-72.0%)<br><br>COVID-19 Omicron hospitalization for 2-dose ChAdOx1 nCoV-19 in 18-19 years.

Name	Country	Study design	Age	Sample size (cases/total)	Vaccination type	Time	Main outcome
							VE (no time limit) = 53.0% (12.0%-75.0%)<br><br>COVID-19 Omicron hospitalization for 1-dose CoronaVac in 18-19 years.<br><br>VE (no time limit) = 26.0% (-25.0%-68.0%)<br><br>COVID-19 Omicron hospitalization for 2-dose CoronaVac in 18-19 years.<br><br>VE (no time limit) = 44.0% (11.0%-65.0%)<br><br>COVID-19 Omicron severe infection for 1-dose BNT162b2/ChAdOx1 nCoV-19/CoronaVac in 12-19 years.<br><br>VE (no time limit) = 23.0% (-17.0%-49.0%)<br><br>COVID-19 Omicron severe infection for 2-dose BNT162b2/ChAdOx1 nCoV-19/CoronaVac in 12-19 years.<br><br>VE (no time limit) = 25.0% (-18.0%-49.0%)
González 2022	Argentina	Cohort study	3-17 years	1536435/1536435	2-dose BBIBP-CorV vaccine<br><br>2-dose vaccination	No time limit	COVID-19 Omicron hospitalization for 2-dose BBIBP-CorV in 3-11 years.<br><br>VE (no time limit) =58.6% (4.1%-79.7%)<br><br>COVID-19 Omicron severe infection for 2-

Name	Country	Study design	Age	Sample size (cases/total)	Vaccination type	Time	Main outcome
							dose vaccination in 12-17 years.<br><br>VE (no time limit) =78.2% (42.0%-90.3%)
Jara 2022	Chile	Cohort study	3-5 years	194427/490694	2-dose CoronaVac vaccine	No time limit	COVID-19 Omicron hospitalization for 2-dose CoronaVac vaccine in 3-5 years.<br><br>VE (no time limit) =38.2% (36.5%-39.9%)<br><br>COVID-19 Omicron symptomatic infection for 2-dose CoronaVac vaccine in 3-5 years.<br><br>VE (no time limit) = 64.6% (49.6%-75.2%)<br><br>COVID-19 Omicron severe infection for 2-dose CoronaVac vaccine in 3-5 years.<br><br>VE (no time limit) = 69.0% (18.6%-88.2%)
Jang 2023	Korea	Case-control study	5-11 years	29473/3046386	2-dose BNT162b2 mRNA vaccine	15-30 days<br><br>31-60 days<br><br>61-90 days<br><br>1-90 days	COVID-19 Omicron symptomatic infection for 2-dose BNT162b2 mRNA in 5-11 years.<br><br>VE (15-30 days) = 57.6% (51.6%-62.8%)<br><br>VE (31-60 days) = 46.9% (43.7%-49.9%)<br><br>VE (61-90 days) = 41.2% (34.3%-47.4%)<br><br>COVID-19 Omicron severe infection for 2-

**Name**	**Country**	**Study design**	**Age**	**Sample size (cases/total)**	**Vaccination type**	**Time**	**Main outcome**
							dose BNT162b2 mRNA in 5-11 years.<br><br>VE (1-90 days) =100% (100%-100%)

VE: Vaccine effectiveness; COVID-19: coronavirus disease 2019.

Supplemental Table 2. Quality of evidence in included studies.

Study	Newcastle-Ottawa scale score			Total score	Study quality
	Selection	Comparability	Outcome /Exposure		
Amir 2022	3	2	2	7	High
Buchan 2022	3	1	2	6	Moderate
Castelli 2022	4	1	2	7	High
Chemaitelly 2022	4	2	2	8	High
Chiew 2022	4	1	2	7	High
Cocchio 2022	4	1	1	6	Moderate
Cohen-Stavi 2022	4	1	1	6	Moderate
Dorabawila 2022	4	1	1	6	Moderate
Fleming-Dutra 2022	3	1	2	6	Moderate
Florentino 2022	4	1	2	7	High
Florentino' 2022	4	1	2	7	High
Florentino" 2022	4	1	2	7	High
Fowlkes 2022	4	0	2	6	Moderate
González 2022	4	1	2	7	High
Jang 2023	4	1	2	7	High
Jara 2022	4	2	1	7	High
Klein 2022	4	0	1	5	Moderate
Leung 2023	4	0	1	5	Moderate
Nordström 2022	4	2	1	7	High
Oliveira 2022	4	2	2	8	High
Powell 2022	4	0	1	5	Moderate
Price 2022	3	1	1	5	Moderate
Risk 2022	4	1	2	7	High
Rosa 2022	4	0	1	5	Moderate
Rudan 2022	4	1	2	7	High
Sacco 2022	4	0	1	5	Moderate
Saito 2022	4	0	1	5	Moderate
Simmons 2022	4	2	1	7	High
Tan 2022	4	1	1	6	Moderate
Tartof 2022	4	1	2	7	High
Tsang 2022	4	2	2	8	High
Wang 2022	3	1	1	5	Moderate
Wanlapakorn 2022	3	1	1	5	Moderate
